# Clinical decision support for high-cost imaging: A randomized clinical trial

**DOI:** 10.1371/journal.pone.0213373

**Published:** 2019-03-15

**Authors:** Joseph Doyle, Sarah Abraham, Laura Feeney, Sarah Reimer, Amy Finkelstein

**Affiliations:** 1 Sloan School of Management, Massachusetts Institute of Technology, Cambridge, Massachusetts, United States of America; 2 Department of Economics, Massachusetts Institute of Technology, Cambridge, Massachusetts, United States of America; 3 Abdul Latif Jameel Poverty Action Lab, Department of Economics, Massachusetts Institute of Technology, Cambridge, Massachusetts, United States of America; 4 Aurora Health Care, Milwaukee, Wisconsin, United States of America; Public Library of Science, UNITED KINGDOM

## Abstract

There is widespread concern over the health risks and healthcare costs from potentially inappropriate high-cost imaging. As a result, the Centers for Medicare and Medicaid Services (CMS) will soon require high-cost imaging orders to be accompanied by Clinical Decision Support (CDS): software that provides appropriateness information at the time orders are placed via a best practice alert for targeted (i.e. likely inappropriate) imaging orders, although the impacts of CDS in this context are unclear. In this randomized trial of 3,511 healthcare providers at Aurora Health Care, we study the impacts of CDS on the ordering behavior of providers. We find that CDS reduced targeted imaging orders by a statistically significant 6%, however there was no statistically significant change in the total number of high-cost scans or of low-cost scans. The results suggest that the impending CMS mandate requiring healthcare systems to adopt CDS may modestly increase the appropriateness of high-cost imaging.

## Introduction

There is widespread concern in the medical profession and among payers over the health risks and healthcare costs from the inappropriate use of high-cost imaging such as CT scans and MRIs.[1–4] Estimates suggest that as much as 30% of diagnostic imaging in the U.S. is unnecessary.[5–7] In 2014, Medicare enrollees received on average about 0.9 high-cost scans, and Medicare spending on high-cost imaging was over $4 billion.[8,9] Total Medicare costs from high-cost imaging are likely considerably greater, due to the additional follow-up care such scans can trigger.[2,10]

Reflecting these concerns, in 2014 the Centers for Medicare and Medicaid (CMS) announced that, in order to be reimbursed by Medicare, high-cost images will have to be ordered through a Clinical Decision Support (CDS) system that meets specific Appropriate Use Criteria (AUC).[11] CDS is typically built into the existing order entry system and provides information to healthcare providers about scan appropriateness at the time of ordering. The CMS requirement is currently slated to go into effect January 1, 2020.[12]

Observational studies of CDS for high-cost imaging have produced mixed evidence on effectiveness. For example, a number of pre-post studies have found that the roll-out of CDS was correlated with 50–80% reductions in scans deemed likely inappropriate; [13–18], and some evidence of a reduction in CT scans for specific injuries such as head injury and c-spine injury [19], however the high-profile Medicare Imaging Demonstration project found little to no impact.[20] Moreover, in these studies it is difficult to determine whether effects are due to the introduction of CDS per se, to contemporaneous efforts by hospitals to alter high-cost scanning procedures, or to underlying secular trends. One study randomized access to decision support rules in the setting where providers responded to vignettes and the decision to order a brain CT scan, which confirmed the prospect of large impacts of CDS [21]. S1 Appendix provides additional detail on the existing literature.

The overall aim of the project was to conduct and analyze the first randomized evaluation of the impact of any CDS system on high-cost imaging in practice to respond to calls for such evidence [7,22,23] and to inform the impending CMS mandate. On December 15, 2016, Aurora Health Care—the largest healthcare system in Wisconsin—successfully launched a prospective, randomized controlled trial of a leading, CMS-compliant CDS for high-cost imaging by introducing it to a randomly selected half of its healthcare providers. The other half continued under the existing order entry system, and the trial lasted for one year as planned. This provider-level randomized design provides the opportunity to study the impact of CDS on imaging over the first year of its introduction.

## Materials and methods

### Study design

This trial is an investigator-initiated, randomized study. The study received IRB approval from Aurora (IRB #s 15-136E, and 15-138E) on December 2, 2016, and the study period began on December 15, 2016. The trial was submitted for registration on December 14, 2016 at clinicaltrials.gov (NCT02996045) [24] and the American Economic Association (RCT ID: AEARCTR-0001846) [25]. The clinicaltrials.gov protocol was first posted on December 19, 2016, four days after the start of the trial due to delays in finalizing the formatting of the registration, with no substantive changes from the original submission. For the considerations of informed consent, the IRB considered two types of subjects: providers randomized as part of the evaluation and patients treated by these providers. The IRB approved a process where the providers were informed about the study by email and had three weeks to opt out of the study. Written consent was not required because the intervention was deemed minimal risk to the subjects. The IRB granted a waiver of informed consent by patients, determining that the research posed no more than minimal risk, that it would not be practicable without the waiver, and that the waiver would not adversely affect the rights and welfare of patients. The trial protocol was written and designed by the authors. Aurora providers, including the co-author, were blinded from interim results during the trial.

**Fig 1:**
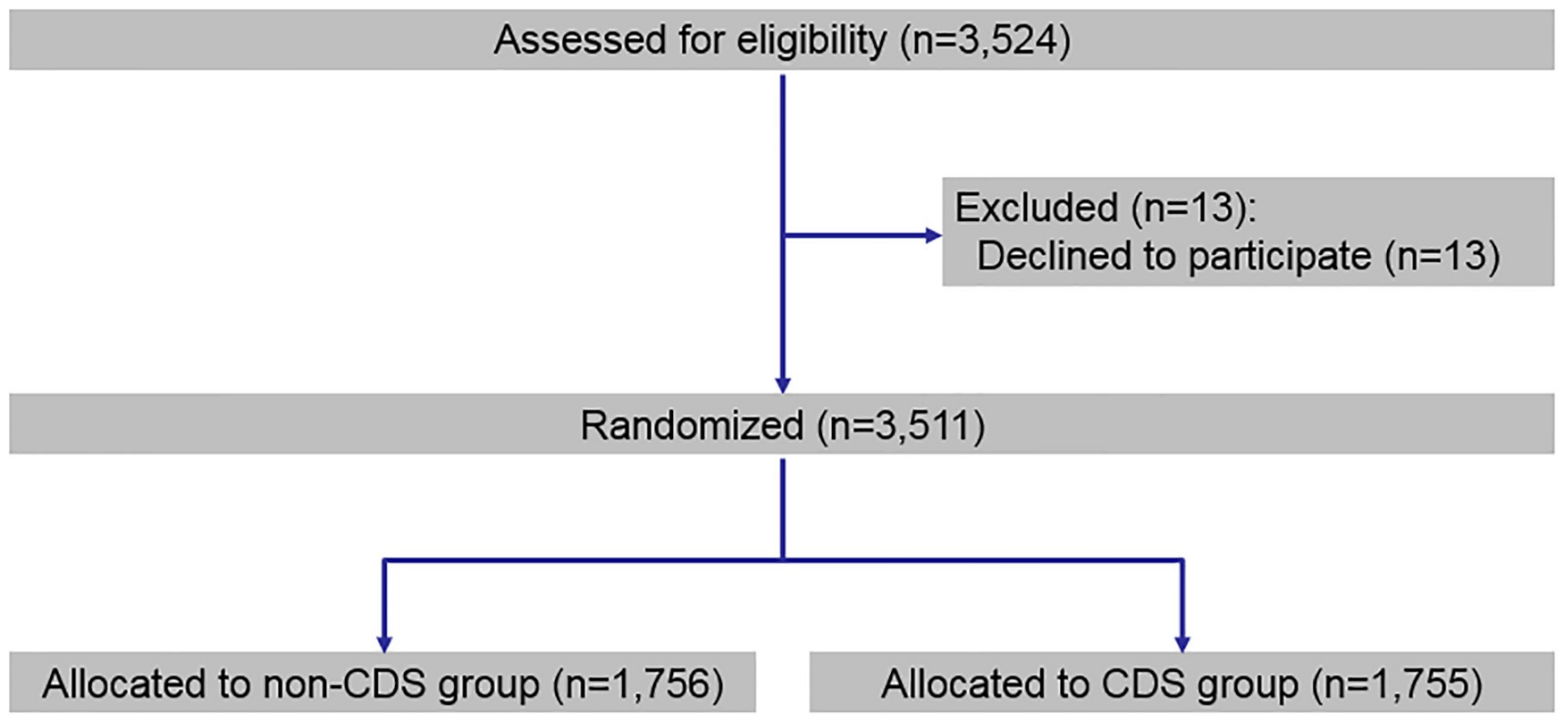
CONSORT flow chart.

Eligible providers for the study included all medical doctors, nurse practitioners, physician assistants, doctors of osteopathy or podiatry, and certified nurse midwives who had at least one imaging study from November 1, 2015 to October 19, 2016, or were a medical resident as of November 2, 2016. Fig 1 below shows the CONSORT flow chart, which shows our provider-level randomization where the treatment group was provided CDS in an environment that would be compliant with the impending CMS mandate, while the control group continued to order as they had prior to when the trial began. The intervention and the randomization protocol are described in detail below.

### CDS intervention

In our study, we randomly assigned providers to receive best practice alerts (BPAs) which display as a pop-up window for providers when they order a likely inappropriate high-cost imaging order and suggest potentially more appropriate alternative orders. BPAs rely on the current ordering environment as a source of information so as to minimize hassle costs, but are highly customizable in the frequency with which they appear and the content they show to providers. Below we detail in turn the software used to produce BPAs, how it integrates with the current ordering process to determine appropriateness, and the form of the BPA we study in our intervention.

#### Software

The CDS tool used by Aurora for radiology is ACR Select. As of November 2017, ACR Select was one of nine systems CMS had declared will be in compliance with the CMS mandate.[26] ACR Select was created by the National Decision Support Company (NDSC) to integrate guidelines for high-cost scans largely from the American College of Radiology (ACR) directly into a hospital’s electronic medical record (EMR), including an integration with Epic, the software Aurora uses for its EMR system.

#### Ordering process and appropriateness scoring

The CDS system uses information from the existing work flow for ordering scans to determine the appropriateness of an imaging order. Within the EMR, all providers must take two actions for an imaging order to be placed: the provider must (1) select a scan type (*e*.*g*. “CT Head/Brain”) and (2) select a structured clinical indication (*e*.*g*. “Headache”); the provider cannot enter free text for the indication and is limited to the set of indications permitted for that order type. For high cost imaging orders, the EMR system then sends this information to ACR Select (the CDS system), including the scan, indication, patient age, and patient gender to determine the appropriateness rating from 1 to 9, with lower scores indicating the order is likely less appropriate. These scores have been agreed upon by an ACR committee consensus for each indication and imaging order pairing. Appropriateness scores are automatically generated for all providers (treatment and control) but never revealed to control providers in any form. The scoring process usually imposes a time delay of 1–2 seconds but never more than 7 seconds within the ordering process.

The CDS system uses the appropriateness scores of the imaging order and alternative orders for the same structured indication to construct a best practice alert (BPA) that provides information to the providers before the order is completed. The logic for when a BPA is shown is primarily based on the score of the order and considered alternatives, but can also include filters for physician types and locations. A pop-up window for the BPA provides the score of the ordered scan as well as a list of alternatives with their scores. The healthcare institution can further customize the BPA to include more information (such as radiation or cost of suggested orders) and can vary in terms of the hassle it imposes (i.e. it could default to cancel a low scoring order). Below we detail the exact logic, content, and format of the BPA we study.

Not all high-cost scans are scored by a CDS system; if they are not scored, they are also not eligible for a BPA. In the large-scale Medicare Imaging Demonstration (MID) study, which found little impact of CDS, about two-thirds of targeted high-cost scan orders were not subject to the CDS; this is believed to have substantially limited its effectiveness.[20,27] Aurora therefore took a number of steps to ensure that scans covered by the CDS were in fact being scored, including prohibiting providers from entering a free text indication. Less than one-quarter of high-cost scans were still not scored by CDS for a variety of reasons; for example, some indications or procedures are not in the set of rules used by ACR Select. Section III.3 in S1 Appendix provides more detail.

#### Logic for targeting imaging orders

**Fig 2:**
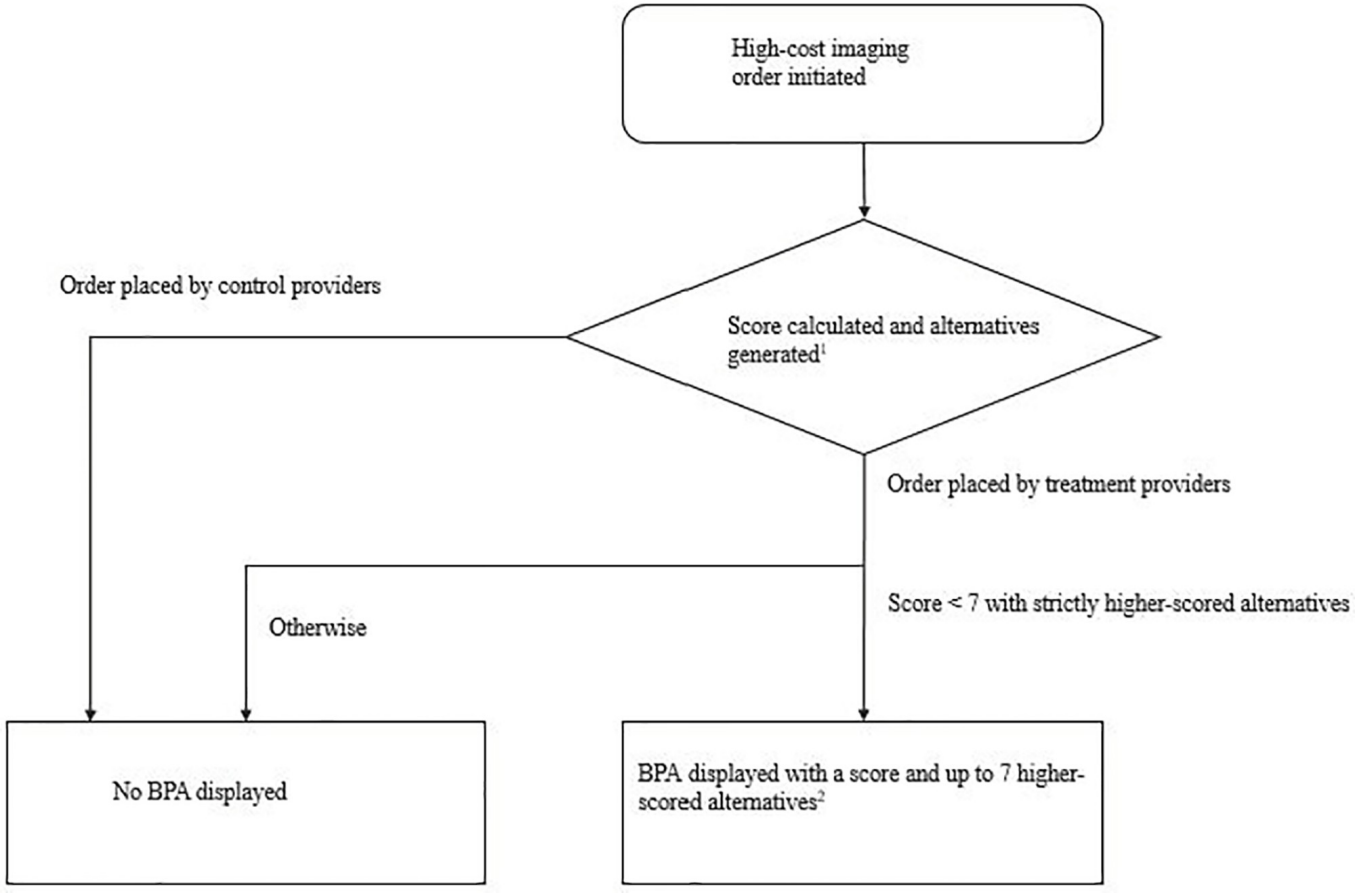
Logic for showing best practice alerts (BPAs). ^1^ Scores are calculated based on a structured indication for the patient’s health problem, a type of image, and patient information including patient age and sex. ^2^ Only alternatives with a score > 4 will be shown.

Fig 2 describes the intervention. Aurora chose to show a best practice alert (BPA) to the treatment group if the CDS system scored the imaging order lower than 7 and had a strictly higher-scoring alternative; we refer to these as “targeted scans.” For the first four months of the trial, a BPA would also be shown for a scan scored 7 or 8 with a strictly higher-scoring alternative and for a scan scored less than 7 with no strictly higher alternatives; in S1 Appendix we show the timeline, as well as results that are similar if we define targeted scans using this alternative logic. When a treatment provider orders an imaging study that meets the above score-based logic criteria, a BPA is shown as a pop-up window displaying the appropriateness score of the request, and the score of up to seven alternatives with scores greater than 4 and greater than or equal to the original request’s score. Options are color coded red (for likely inappropriate scores 1–3), yellow (4–6), or green (likely appropriate, 7–9).

**Fig 3:**
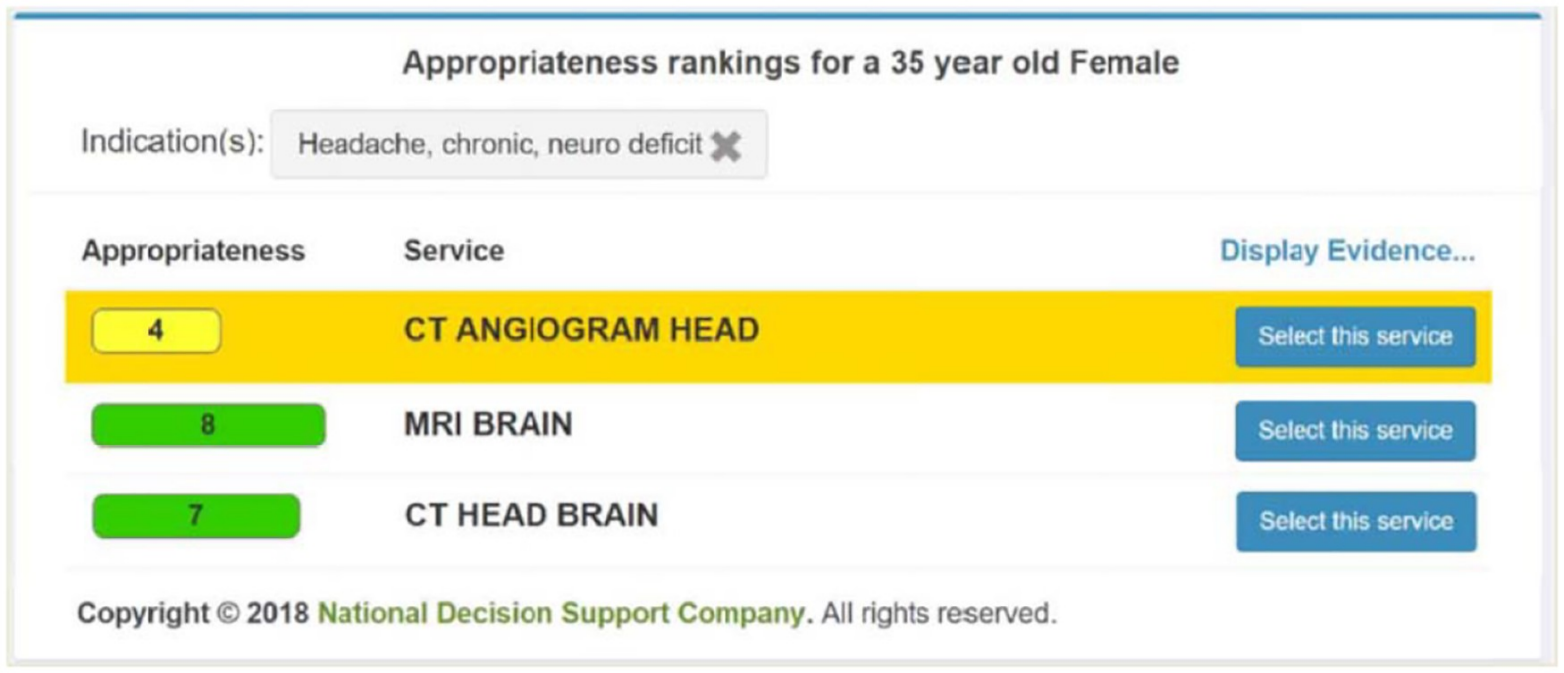
Example, best practice alert (BPA). Note: Reprinted under a CC BY license, with permission from the National Decision Support Company, original copyright 2018.

Fig 3 shows an example of the content of the BPA. Within the BPA, providers could click on a hyperlink to read more about the evidence behind the recommendations from the American College of Radiology; providers could also continue with the original order, cancel the order, or replace the order with a suggested alternative. In practice, all of those action items were available in a panel below the graphic shown in Fig 3 as part of the order entry system. 97% of BPAs shown suggest alternatives. However, the BPA defaults to keep the original order unless the provider actively selects an alternative.

### Study population and procedures

The study population was drawn from Aurora providers who ordered at least one imaging study (including low-cost imaging studies) from November 1, 2015 to October 19, 2016, and additionally included any medical residents at Aurora as of November 2, 2016 when the recruitment email was sent. We limited the set of eligible providers to medical doctors, nurse practitioners, physician assistants, doctors of osteopathy or podiatry, and certified nurse midwives, to focus on the target population for decision-making software. We excluded nurses and clerical workers from the study population even though, as we discuss below, they may order scans on behalf of study providers.

Fig 1 shows the study flow chart. Of the 3,524 eligible providers informed of the study 13 providers opted out and received CDS but were excluded from the study. We randomly assigned half of the remaining 3,511 providers to receive CDS (“CDS group” or “treatment group”) while the other half continued to order as usual (“control group”).

Both groups continued to use the status quo order entry system, which did not change with the introduction of CDS. Treatment-group providers began receiving BPAs for orders targeted by the CDS system so that their environment became compliant with the impending CMS mandate. In contrast, control-group providers continued to order as they had before the trial began: they received no feedback about appropriateness scores through BPAs or any other means. Scores for the orders placed by control-group providers were calculated for research purposes only.

### Data and outcome measures

All analyses were based on administrative data supplied by Aurora Health and NDSC. NDSC data include scan-indication orders, scores, and scores of alternatives and are collected for both the treatment and control groups even though the control group was not shown any scoring information. Aurora data include patient encounters and demographics, provider characteristics, and scan-indication orders. We study all scans ordered by study providers, except those for patients under 18 or ordered during behavioral-health encounters, as requested by Aurora’s IRB.

All outcomes were measured over our 12-month study period (December 15, 2016 –December 15, 2017). Baseline data for treatment and control providers were collected over an 8-month “quiet period” (April 22 2016 –December 14 2016), during which imaging orders and alternatives were scored, but the BPA could never be shown.

Our primary outcome was the number of orders for targeted scans–i.e. orders for which a BPA would be shown if the CDS were in place. Declines in targeted scans could reflect clinically relevant changes in patient treatment, or merely “indication gaming”–i.e. changes in coded indications; for example, changing the indication for a CT scan of the head from “headache” to “thunderclap headache” would increase its score from 3 to 9 thereby making it no longer a “targeted scan”.

Secondary outcomes included the number of orders for sub-categories of targeted scans: CT scans, MRI scans, orders scored 1–3 (“red orders”), and orders scored 4–6 (“yellow orders”). Secondary outcomes also included the total number of high- and low-cost orders. High-cost orders consist of CT, MRI, nuclear medicine, and PET; low-cost orders consist of x-ray, ultrasound, mammogram, fluoroscopy, and bone densitometry. We also examined heterogeneity in the impact of CDS across subgroups of providers and over time, although this exploratory analysis was not pre-specified due to a lack of statistical power.

Prior to the start of the trial, we pre-specified the primary outcome and all of the secondary outcomes except for the split into targeted CT scans and targeted MRI scans [24,25]. Post hoc heterogeneity analyses and balance tests were not pre-specified, since complete data on provider characteristics were not available prior to the trial start date.

### Statistical analyses

Based on historical data available before the trial, we estimated that with the sample size in this trial we had 80 percent power to detect a 13 percent reduction in our primary outcome [24,25] (2-sided size = 0.05).

To compare the two groups at baseline, two-tailed t-tests of the equality of the two means, and an F-test of the equality of means across all characteristics, were calculated using heteroskedasticity-robust standard errors.

Our main intent-to-treat analysis used linear regressions to compare behavior of treatment and control providers; as pre-specified [24,25], to increase precision we controlled for the value of the dependent variable in the quiet period. Our results are robust to a variety of alternative specifications (see V.1 in S1 Appendix). All statistical analyses, including the randomization, were conducted using STATA version 13.

## Results

### Balance of providers

Characteristics of providers were balanced across treatment and control groups (Table 1); an F-test failed to reject equality of all baseline measures (p-value = 0.44). About two-thirds of providers were medical doctors. During the quiet period, about two-thirds of providers had their modal patient encounter in an outpatient setting, with the rest split between inpatient and ED.

**Table 1:** Provider characteristics.

	Control Group, (n = 1,756)	CDS Group, (n = 1,755)	P-Value^a^
Provider age as of December 15, 2016, mean (SD)	46.5 (11.6)	46.3 (11.5)	0.61
Men, No. (%)	1,003 (57.1)	990 (56.4)	0.67
Years since graduation from medical school, mean (SD)	22.4 (10.9)	22.3 (10.7)	0.96
Missing graduation date, No. (%)	556 (31.7)	523 (29.8)	0.23
Provider type, No. (%)			
Medical Doctor	1,118 (63.7)	1,174 (66.9)	0.04
Doctor of Osteopathy	126 (7.2)	108 (6.2)	0.23
Doctor of Podiatric Medicine	20 (1.1)	20 (1.1)	1.00
Nurse Practitioner	271 (15.4)	244 (13.9)	0.20
Physician Assistant	204 (11.6)	191 (10.9)	0.49
Certified Nurse Midwife	17 (1.0)	18 (1.0)	0.86
Primary encounter location during Quiet Period, No. (%)^b^			
Hospital, non-ED	251 (14.3)	259 (14.8)	0.70
Outpatient setting	1,188 (67.7)	1,194 (68.0)	0.81
Emergency department	317 (18.1)	302 (17.2)	0.51
Imaging orders during Quiet Period (Annualized), mean (SD)^b^			
Number of targeted scans	17.7 (34.2)	17.0 (31.8)	0.56
Number of high-cost scans	108.0 (184.5)	108.2 (181.0)	0.98
Characteristics of patients seen during Quiet Period, mean (SD)^b^			
Average age	55.9 (12.0)	56.5 (11.9)	0.12
Share male	0.41 (0.20)	0.42 (0.20)	0.21
P-Value from F-statistic for balance			0.436

Abbreviations: CDS, clinical decision support; ED, emergency department.

^a^ P-values are calculated for two-tailed t-tests of the equality of the two means, using heteroskedasticity-robust standard errors.

^b^ Quiet Period covers the period 4/22/2016 to 12/14/2016 during which the BPA was not yet firing.

### Impact of CDS

Table 2 shows our main results. Control providers averaged 17 targeted scans during the study period. CDS reduced targeted scans by 1.1 per affected provider (95% CI -2.11 to -0.13; p-value = 0.03). This represents about a 6% decline in our primary outcome relative to the control group over the year of the intervention.

**Table 2:** Impact of CDS on scans.

	Control Group, Mean (SD) (n = 1,756)	CDS Group, Mean (SD) (n = 1,755)	Adjusted Between-Group Difference (95% CI)^a^	P-Value^a^
**Outcome**				
Targeted scans	17.0 (32.6)	15.3 (30.0)	-1.12 (-2.11 to -0.13)	0.027
CT scans^b^	10.5 (24.7)	9.8 (22.4)	-0.94 (-1.63 to -0.25)	0.008
MRI^b^	5.1 (13.5)	4.4 (14.1)	-0.11 (-0.60 to 0.39)	0.676
Red orders (scored 1–3)	5.9 (13.6)	5.3 (12.2)	-0.40 (-0.89 to 0.09)	0.110
Yellow orders (scored 4–6)	11.1 (23.0)	10.1 (20.9)	-0.76 (-1.48 to -0.04)	0.039
All High-cost scans	108.3 (188.7)	106.7 (179.3)	-1.74 (-5.90 to 2.42)	0.412
All Low-cost scans	370.1 (598.8)	333.3 (445.9)	-9.38 (-21.76 to 3.01)	0.138

Abbreviations: CDS, clinical decision support; CT, computed tomography; MRI, magnetic resonance imaging.

^a^ Effect of CDS was estimated from a linear regression of the outcome on an indicator for whether the ordering provider was a treatment provider. All regressions include a control for the lag of the dependent variable; p-values and confidence intervals are based on heteroskedasticity-robust standard errors.

^b^ Outcome not prespecified in trial registry.

CDS reduced targeted CTs by 0.94 (95% CI -1.63 to -0.25; p-value = 0.01) or by 9% relative to the control group, and the difference in targeted MRIs was not statistically significant: a reduction of 0.11 (95% CI -0.60 to 0.39; p-value = 0.68), or 3% relative to the controls. CDS reduced targeted red orders by 0.40, which is not statistically significant at the 5% level (95% CI -0.89 to 0.09; p-value = 0.11), and targeted yellow orders by 0.76 (95% CI -1.48 to -0.04; p-value = 0.04); both represent 7% declines relative to the control.

CDS reduced total high-cost scans in annual levels by 1.74 (95% CI -5.90 to 2.24; p-value = 0.42), and total low-cost scans by 9.4 (95% CI -21.76 to 3.01; p-value = 0.14); both represent 2% declines relative to the control and are not statistically significant.

Table 3 explores heterogeneity in the impact of CDS on targeted scans. These analyses were not pre-specified, warranting some caution in the interpretation. Although differences across subgroups are not statistically significant even without correcting for multiple hypothesis testing, differences in point estimates can suggest the sources of the main results and stimulate further research into the types of providers impacted by CDS. Panel A compares providers who vary in their volume of ordering, which affects the frequency with which they are shown BPAs and may be related to the provider’s knowledge of the imaging guidelines. Among providers in the bottom quartile of targeted scan orders in the eight months prior to the intervention, CDS reduced targeted scans by about 36% (point estimate -0.25, 95% CI -0.050 to -0.00; p value = 0.05) while for those in the top quartile, CDS reduced targeted scans by about 7% (point estimate -3.56, 95% I -7.28 to 0.16; p-value = 0.06).

**Table 3:** Subgroup analysis of impact of CDS on targeted scans^a^.

	Control Group, Mean (SD) (n = 1,756)	CDS Group, Mean (SD) (n = 1,755)	Adjusted Between-Group Difference (95% CI)^b^	P-Value^b^
**Panel A: Quartile of targeted scan orders in the quiet period**^c^				
1	0.7 (2.2)	0.5 (2.3)	-0.25 (-0.50 to 0.00)	0.046
2	3.8 (4.9)	3.2 (5.6)	-0.59 (-1.56 to 0.38)	0.230
3	10.4 (8.8)	10.3 (8.6)	-0.45 (-1.46 to 0.56)	0.383
4	53.7 (47.2)	47.8 (44.9)	-3.56 (-7.28 to 0.16)	0.061
**Panel B: Quarter of study period**^c^				
1	4.3 (8.5)	4.0 (8.0)	-0.18 (-0.45 to 0.10)	0.206
2	4.4 (8.8)	3.9 (7.9)	-0.33 (-0.62 to -0.04)	0.025
3	4.2 (8.6)	3.7 (7.7)	-0.37 (-0.68 to -0.06)	0.021
4	4.1 (8.4)	3.8 (8.0)	-0.25 (-0.57 to 0.08)	0.138
**Panel C: Scan Order Location**				
Hospital, non-ED	1.1 (5.7)	1.1 (6.8)	0.05 (-0.26 to 0.35)	0.762
Outpatient Setting	5.5 (14.3)	4.6 (12.2)	-0.48 (-0.90 to -0.05)	0.028
Emergency Department	7.3 (25.0)	6.9 (22.7)	-0.54 (-1.16 to 0.08)	0.089
Locations not in-person^d^	3.0 (7.6)	2.7 (7.4)	-0.24 (-0.49 to 0.02)	0.071
**Panel D: Provider Type**^c^				
Medical Doctor	17.5 (33.5)	16.0 (30.4)	-1.15 (-2.33 to 0.03)	0.057
Doctor of Osteopathy	29.8 (50.6)	22.5 (40.3)	-2.40 (-6.27 to 1.47)	0.223
Nurse Practitioner	9.6 (18.1)	10.5 (27.0)	0.72 (-2.15 to 3.58)	0.624
Physician Assistant	18.4 (27.5)	15.5 (25.9)	-2.08 (-5.35 to 1.20)	0.214
Other^e^	4.2 (12.0)	5.0 (11.6)	0.73 (-0.93 to 2.40)	0.382
**Panel E: Provider Age**^c^				
Below Median	20.0 (37.3)	17.5 (32.1)	-1.51 (-3.19 to 0.17)	0.078
Median or Above	14.3 (27.5)	13.4 (27.9)	-0.77 (-1.88 to 0.34)	0.173

Abbreviations: CDS, clinical decision support; ED, emergency department.

^a^ All analyses are for primary outcome (targeted scan).

^b^ Effect of CDS was estimated from a linear regression of the outcome on an indicator for whether the ordering provider was a treatment provider. All regressions include a control for the lag of the dependent variable; p-values and confidence intervals are based on heteroskedasticity-robust standard errors.

^c^ Subgroup analysis not prespecified in trial registry.

^d^ Targeted scans not ordered during in-person encounters were ordered by telephone (60%), as an order without an encounter (24%), or within other clinical scenarios (16%).

^e^ Includes podiatrists (53%) and midwives (47%), as described on Table 1.

Next we considered whether the effectiveness varies over time. Panel B indicates that the impact of CDS was roughly similar across all four quarters of the study period. Our study setting includes providers across a range of practice settings, and Panel C shows that the estimated effect is small and of opposite sign in the inpatient setting. The finding of similar declines in targeted scans in the ED setting and outpatient setting is reassuring, since it suggests that decision making in teams (which could include treatment and control providers) is not reducing our effect; such team-based decision is more common in the ED than in the outpatient setting. In addition, Panels D and E show no evidence of differential impacts of CDS by provider type or provider age.

In section V.2 in S1 Appendix we tested whether the distribution of indications on ordered high-cost scans was the same for treatment and control providers, and could not reject the null of no difference in distribution (p-value = 0.56), which is consistent with a lack of “indication gaming”. We also tested whether, in response to the intervention, scan orders were directed away from treatment providers to providers who would not trigger a BPA. This could happen if some patients were sent to control providers instead of treatment providers, if control providers entered some scan orders on behalf of treatment providers, or if treatment providers were more likely than control providers to have their scan ordered by personnel (such as nurses) whose entries would not trigger a BPA; we found no evidence of effects.

## Discussion

This randomized trial of 3,511 healthcare providers examined the effect of one of the market leaders in radiology CDS, an application that is compliant with the CMS mandate that high-cost images be ordered through CDS in order to be reimbursed by Medicare. CDS reduced targeted scans–i.e., the scans it was designed to reduce–by a statistically significant 1.1 orders per affected provider per year. Still, this represented only a 6% reduction relative to the control group, leaving the vast majority of targeted scans still extant. CDS had a more limited impact on the overall number of high-cost images ordered. One reason for such a muted response is that CDS often makes a recommendation for another high-cost imaging order: for an average provider in our sample, 69% of BPAs contain at least one high-cost image as an alternative.

The reduction in targeted scans persisted over the entire one-year study period, with no evidence of “alert fatigue” reducing effectiveness over time. CT scans—which are the most-common high-cost imaging type and carry the greatest concerns about over-ordering [28]—were responsible for four-fifths (0.94/1.12) of the overall reduction in targeted scans. CDS had no statistically significant effect on total high-cost scans, which is not surprising since it is designed to reduce “inappropriate” high-cost scans, not all high-cost scans. There was no evidence of ‘indication gaming”, consistent with findings in the prior literature.[16,17,27,29,30]

### Limitations

This study has a number of limitations. First, our test of “indication gaming” was limited, so it is possible that some of the decline in targeted scans did not reflect clinically relevant changes in patient treatment. Gaming itself can be limited by restricting the set of indications that are available for a particular scan type, but we did not have variation to explore this alternative option for encouraging more appropriate scans. Second, our study took place at one particular institution–a large, non-profit healthcare system operating throughout eastern Wisconsin with 15 hospitals and more than 150 clinics–which may have particular types of patients and providers. The impact of CDS may vary in other settings.

Third, our estimates speak to the impact of a particular CDS system; other configurations could have more or less of an effect. For example, organizations can change the rules that govern how often the BPA is shown, whether the BPA provides information on scan costs or radiation, or whether the CDS defaults to place an order for the highest scoring alternative instead of defaulting to the original order. CDS software can also be combined with other interventions. Since a goal of our study was to isolate the effect of showing BPAs to providers based on CDS scores, we did not study other interventions which are enabled by the CDS scoring system, such as supervisor oversight or public reporting of provider ordering behavior; these are conjectured to be an important aspect of maximizing the impact of CDS.[31]

Fourth, it is not straightforward to connect order requests and the range of alternatives provided to changes in ordering at the time of a BPA encounter due to data limitations. This is partly because providers rarely change their orders within the BPA. Instead, they cancel an order and then make a new request. In any event, our estimated effects stem from both the direct effect of providers responding to information in a BPA at the time of ordering and the indirect effect of providers learning from BPAs over time and reducing inappropriate order requests.

Fifth, the study only considered the impact of CDS on imaging orders. A more complete analysis would also examine CDS impacts on downstream healthcare utilization, patient health, and provider perceptions of the burden or benefits of CDS.

## Conclusion

Despite these limitations, this study provides what is, to our knowledge, the first large-scale randomized trial of the impact of CDS on high-cost imaging. The results demonstrate that a software intervention alone can significantly reduce targeted high-cost imaging. While some pre-post comparisons of CDS implementation has found large reductions in imaging orders, our findings show that the vast majority of targeted scans were not eliminated. Given concerns about the cost and health implications of inappropriate high-cost imaging–as well as the imminent CMS mandate that CDS be used for high-cost imaging to be eligible for Medicare reimbursement—an important area for further work is to rigorously evaluate what design features of CDS can increase its effect.

## Supporting information

S1 Appendix Supporting information. (DOCX)

S1 CONSORT Checklist (DOC)

S1 Trial Protocol Study protocol and Statistical analysis plan. (DOCX)
