# Supplementary material for: Clinical decision support for high-cost imaging: A randomized clinical trial
Source: PLoS One. 2019 Mar 15;14(3):e0213373. doi: 10.1371/journal.pone.0213373 (PMC6419998; doi:10.1371/journal.pone.0213373)
Supplement: S1 Appendix — (DOCX) [file pone.0213373.s001.docx]

S1 Supporting Information

Contents

[I. Existing research on CDS 3](#_Toc511207111)

[II. Study Design 4](#_Toc511207112)

[II.1 Setting and Timeframe 4](#_Toc511207113)

[II.2 Clinical Decision Support (CDS) Intervention 5](#_Toc511207114)

[ACR guidelines and ACR Select 5](#_Toc511207115)

[Order Work Flow 5](#_Toc511207116)

[CDS logic for showing the BPA 6](#_Toc511207117)

[III. Data 7](#_Toc511207118)

[III.1. Data Sources 7](#_Toc511207119)

[III.2 Variable Definitions 8](#_Toc511207120)

[III. 3 Summary statistics. 8](#_Toc511207121)

[Distribution of ordering behavior 8](#_Toc511207122)

[Unscored Scans 9](#_Toc511207123)

[IV. Estimating Equation 9](#_Toc511207124)

[V. Additional Results 10](#_Toc511207125)

[V.1 Sensitivity analysis 10](#_Toc511207126)

[V.2 Strategic Avoidance of CDS 10](#_Toc511207127)

[Handoffs to providers not subject to CDS 10](#_Toc511207128)

[Reduced patient volume 11](#_Toc511207129)

[Indication changes 12](#_Toc511207130)

[Figure S1: Project Timeline 1](#_Toc511207131)4

[Table S1: Distributional Statistics on Orders Placed by Control Providers 1](#_Toc511207133)5

[Table S2: Types of Imaging Orders Placed by Control Providers 16](#_Toc511207134)

[Table S3: Top 20 Most Frequent Indication-Imaging Pairs Ordered By Control Providers 17](#_Toc511207135)

[Table S4: Top 20 Most Frequent Targeted Indication-Imaging Pairs Ordered By Control Providers 18](#_Toc511207136)

[Table S5: Impact of CDS on Scores of Imaging Orders 19](#_Toc511207137)

[Table S6: Sensitivity analysis for Impact of CDS on Primary Outcome (Targeted Scans) 20](#_Toc511207138)

[Table S7: Analysis of Potential CDS Avoidance 2](#_Toc511207139)1

[VI. References 2](#_Toc511207140)2

This Appendix provides additional information on the existing literature, as well as additional details on the study population, data and variable construction, empirical strategy, and results.

# Existing research on CDS

Despite the concerns about over-scanning, the interest in CDS as a way to mitigate this problem, and the upcoming policy change requiring use of CDS for Medicare reimbursement of high-cost imaging, we know of no large-scale randomized trials on the impact of CDS for imaging. By contrast, there are a number of RCTs for decision support tools designed to prevent prescribing leading to harmful drug interactions or adverse drug events,^1–4^ reduce redundant laboratory test orders,^5–7^ and encourage appropriate use of medications and laboratory testing.^4,8–14^ A number of reviews of the decision-support literature note the dearth of randomized controlled trial evidence when it comes to tools to support diagnostic imaging.^15–17^

We have been able to identify only one provider-level RCT carried out in a clinical setting that tests the effect of CDS with appropriateness guidelines on diagnostic imaging.^18^ This study was of 66 providers at an urgent care center who were randomized into either CDS for radiography for patients with foot and ankle injuries, or to control; it found that CDS increase adherence to guidelines. Another study conducted an RCT (randomized at the patient level) of providing providers with information on charges for clinical laboratory and radiological tests at the time of the decision; no statistically significant effect was recorded on the number of tests ordered.^19^ We found two others studies that conducted a physician-level RCT where physicians were asked to evaluate vignettes (i.e. hypothetical scenarios), which found that including information about guidelines could affect physician choices diagnostic tests and imaging in these hypothetical scenarios.^20-22^

Observational studies of CDS for high-cost imaging have produced mixed evidence on effectiveness. For example, a number of pre-post studies have found that the roll-out of CDS was correlated with 50-80% reductions in scans deemed inappropriate,^23–28^ and some evidence of a reduction in CT scans for specific injuries such as head injury and c-spine injury^29^. However, not all observational studies have concluded that CDS is effective. For example the high-profile Medicare Imaging Demonstration (MID) project found little impact to no impact on scan ordering.^30^ The MID project included over 5,000 physicians across seven partnering conveners to compare physicians before and after the implementation of a CDS system. One possible contributing factor is that about two-thirds of scans were not scored, either because doctors managed to avoid the CDS or because CDS was not available for the clinical scenario.

The general concern with pre-post studies that it is difficult to assess what would have happened in the absence of the intervention. In the particular context of CDS, there is the added concern that its introduction often coincides with other efforts by the hospital to reduce high-cost scanning procedures. For example, in one study,^23^ pre-authorization of high-cost scans was waived for scans placed through the electronic medical record (EMR); this was associated with to more than a 100% increase in scan volume routed through the EMR after CDS was implemented, and drastically different composition of scans ordered pre- versus post- CDS. Such contemporaneous changes make it difficult to isolate the causal effect of CDS.

# Study Design

## II.1 Setting and Timeframe

Aurora Health Care is a non-profit, healthcare system in Wisconsin. It was founded in 1984 through the merger of two existing Milwaukee hospitals. Currently, it consists of 15 hospitals, with more than 150 clinics and 70 pharmacies throughout eastern Wisconsin. It includes a variety of practice settings, including small clinics, multi-specialty large clinics, suburban hospitals, urban hospitals and quaternary care hospitals. According to the 2014 Provider of Service Data from CMS, Aurora had about 12% of the total number of hospital beds in WI. The Aurora network includes a range of small (60-70 bed) and large (300-400 bed) hospitals. The median-sized hospital in the system is around the national median.

Appendix Figure 1 summarizes the timeline for the study. The CDS went “live” for treatment providers on the morning of December 15, 2016; this was the first day in which the treatment group could be shown best practice alerts (BPAs) if their ordering so warranted. We planned and pre-specified a one year study (through December 15, 2017).

Our **study period** is therefore from December 15, 2016 through December 15, 2017. For much of our analysis we also include ordering behavior during the “pre-study period” – also referred to as the **quiet period** - which covers April 22, 2016 through December 14, 2016. The basic order entry system had been in place prior to April 22, 2016, but it was on April 21, 2016 that a change was made (which persists through our study period) requiring providers to enter a structured indication (i.e. choose from a pre-populated list of indications) rather than having the option to provide a free-text entry as the reason for the scan. During the entire time of the study (and preceding it) the order entry system was supported by National Decision Support Company (NDSC), a third party vendor which provides the software to implement the ruleset and the design of the associated BPA.

Data were de-identified by researchers at Aurora and sent to MIT via a secure FTP. MIT researchers performed the randomization (using STATA 13) on the de-identified list of subjects; the de-identified list of subjects and their treatment status were returned to Aurora, which then relayed the information to NDSC, so that it could turn on the capability of the BPA to be shown to the treatment group but not the control group. The MIT researchers worked with the Aurora team to ensure and verify that this transfer of information was conducted accurately and that the two groups received CDS or not as intended. The MIT authors vouch for analytic accuracy and completeness, as well as fidelity to the study protocol.

## II.2 Clinical Decision Support (CDS) Intervention

### ACR guidelines and ACR Select

The CDS tool that we study, ACR Select, was created by the National Decision Support Company (NDSC) to integrate guidelines largely from the American College of Radiology (ACR) directly into a hospital’s electronic medical record (EMR). Scores given by the CDS are a computerized version of guidelines created by the American College of Radiology (ACR).

The guidelines score the appropriateness of a scan order for a given clinical indication, where indications include common symptoms and diagnosis keywords, such as “acute headache.” In particular, indication-scan pairs are assigned an “appropriateness rating” from 1-9, with lower scores being less appropriate. Scores 1-3 are ‘usually not appropriate,’ 4-6 are ‘may be appropriate,’ and 7-9 are ‘usually appropriate.’ The CDS will then show a “best practice alert” (BPA) as a function of the “appropriateness” score and the appropriateness score of alternative potential scans.

In 1995, ACR published its first set of medical guidelines on imaging and treatment decisions.^31^ Since then, ACR has iterated on the construction of these guidelines, arriving at the current process for deliberation in 2012, in which ACR releases guidelines for diagnostic imaging through its (then newly-formed) Committee on Diagnostic Imaging/Interventional Radiology Appropriateness Criteria.^32^ Over 300 volunteer physicians from a broad range of medical specialties participate in over 20 panels grouped by body system, including subcommittees on radiation exposure and appropriateness criteria methodology, and a committee of panel chairs who make final decisions on appropriateness ratings. Physicians are intentionally selected from diverse geographic regions and from both academic and private practice settings. Each panel selects conditions to be scored based on prevalence, variability of practice, costs, morbidity potential, and potential for improved care. After conditions are selected, the committee conducts a literature search of peer-reviewed journals and ACR staff draft and distribute an evidence table summarizing the selected literature. Physician members then individually score the conditions for a given scan. From these scores, the committee creates a table with a frequency distribution of ratings. If 80% agree about a rating category, then the median rating is assigned as the panel’s final rating. The committee continues this process for up to 3 rounds of ratings, and if no consensus is reached, the committee reports “no consensus” and the scan will not be scored.^32^

### Order Work Flow

The CDS studied here works as follows: To order a high-cost scan from a patient’s chart, the provider must request an imaging order through the radiology order entry system embedded in Epic, the software Aurora uses for its EMR system and one of the industry leaders. Specifically, to order a high-cost scan, the provider must (1) select a scan type (*e.g.* “CT Head/Brain”) and (2) select a structured clinical indication (*e.g.* “Headache”); the provider cannot enter free text for the indication. The EMR system then sends information to a web services platform, ACR Select, including the scan, indication, patient age, and patient gender to determine the appropriateness rating. ACR Select is an industry leader in radiology CDS, whose rule set was developed by the American College of Radiology (as described above). ACR Select scores the order based on the image type, indication(s), and (in some cases) data on patient’s age and gender; if the provider chooses multiple indications, the system selects the maximum score across the image-indication. ACR Select then sends the score back to the EMR along with a list of alternative scans, given the selected indication.

The CDS logic (described below) determines whether or not a Best Practice Alert (BPA) should be shown. The best practice alert (BPA), appears as a “pop-up” within the EMR (for an example, see Figure 3). The CDS displays the appropriateness score as well as more appropriate alternative scan options. By clicking action items at the bottom of the window, providers can proceed with the original order by clicking “accept” or “cancel”, delete their current order, and/or switch to a different scan order. If the provider chooses to make any changes to her order, she must then click “accept” to place the new order. If a BPA is not shown, the order is automatically placed and becomes actionable (i.e. a technician can undertake the scan). Once an order has been placed, the user provider must select an “ordering provider” to sign off on the order; this is defaulted to the user provider if she has clearance to order scans.

The scoring process usually imposes a time delay of 1-2 seconds but never more than 7 seconds within the ordering process. If the delay exceeds 7 seconds the attempt to score the order will be terminated. This occurs for about 0.3% of scans, and can be due to a myriad of reasons but mostly likely due to server downtime on either side or excess traffic on a server.

The above work-flow describes the experience of a treatment provider entering a scan order. We refer to the person entering the scan order into the EMR as the “user provider”. Our analysis is of the behavior of the provider who decides whether a given scan is to be ordered; we refer to this person as the “ordering provider”. The ordering provider is, in most cases, also the user provider. However, it is possible for other personnel to enter orders on behalf of the ordering provider. These other personnel could be other study participants, medical personnel outside of the study such as nurses, or non-medical personnel such as clerical workers and technicians. We refer to cases when the user provider is different from the ordering provider as a “handoff”; the vast majority of handoffs are to nurses. Importantly for our analysis, a treatment ordering provider may not have an order subject to the CDS if it is entered by a user provider who is a control provider, or someone outside of the study. Likewise, a control ordering provider could have an order subject to the CDS if it is entered by a user provider who is a treatment provider. We discuss the implications of such handoffs for interpretation of our findings in Section V.2.

### CDS logic for showing the BPA

ACR Select generates scores 1-9 for many high-cost scan orders; however, not all high-cost scans are scored. We refer to high-cost scans that do not receive a score between 1 and 9 as “unscored”, and discuss them further in Section III.3.

Among high-cost scans that are scored, three factors determine when a BPA is shown: 1) the score of the scan, 2) the score of alternative scans for the selected indication and 3) whether the BPA is repetitive. Aurora precludes “repetitive BPAs”, those that pertain to the same scan-indication pair for the same patient and provider within a 30 minute window.

For the first two criteria Aurora followed more aggressive rules for the first quarter of the study period and slightly reduced alerts for the remainder of the study. From December 15, 2016 to March 29, 2017 the BPA would be shown for treatment providers for an order scored less than 7 or with a strictly higher scoring alternative. For the remainder of the study, the BPA would be shown for treatment providers if the order scored less than 7 *and* have a strictly higher scoring alternative. These criteria created two sets of scans (which represented about 11 percent of scans ordered by the control group in the study period) for which the BPA would be shown for the first four months of the study period but not subsequently: a scan scoring 7 or 8 with a strictly greater alternative, and a scan scoring less than 7 without a strictly higher alternative. Appendix Figure 2 summarizes the BPA logic that was in place for over three-quarters of the study period.

We define our primary outcome **targeted scans** as high-cost scans with a score of less than 7 with a strictly higher scoring alternative. Not all low scoring orders are targeted; the display of the BPA is determined by both the score of the ordered scan and the set of scores for alternative scans for the same indication. During our study period, a BPA would always be shown for targeted scan orders if they were entered by a treatment group provider and were a non-repetitive order; we define targeted scans symmetrically for treatment and control providers.

One idiosyncratic feature of the Aurora-specific ordering process that affects its interaction with the ACR ruleset is that imaging orders placed at Aurora are “contrast generic”; the provider is not required to specify contrast (with, without, or with and without contrast) to complete the imaging order. If the provider did not specify, the radiologist determines how to perform the scan.

According to NDSC, less than 4% of their clients use contrast-generic scoring. The ACR Select ruleset may have different scores for scan-indication pairs with different contrast choices. In a contrast-generic scoring process like Aurora’s for scan-indication pairs with different scores for difference contrast choices, ACR Select chooses the maximum score across these options under the assumption that the provider or radiologist will choose the most appropriate method. This affects the alternatives shown as well as the scoring of the original request. In our study period, 9% of scored high-cost scans ordered by the control group might have had their score affected by contrast generic scoring, and 7% would have had the offered alternatives affected.

# III. Data

## III.1. Data Sources

Our analyses are all based on administrative data collected at Aurora Health and the National Decision Support Company. Historical data were shared in the planning for the study, followed by cumulative monthly data sets shared over the course of the study period.

Our analysis focuses on the types of scans ordered by providers. Our outcome measures are based on scan-request level data from NDSC that contain – for each scan request ordered by one of our study providers – the indication and imaging order requested, as well as the score and set of alternatives. These data allow us to identify the score for the order as well as whether the BPA would be shown if the provider were in the treatment group.

A dataset of ordered scans from Aurora allowed us to link each request observed in the NDSC data to the ordering provider as well as the user provider. We also received an alert-level dataset from Aurora that allowed us to observe which orders showed a BPA, and to confirm that a BPA was shown only when the order was entered by a treatment providers and according to the BPA logic.

Additional data from Aurora supplied us with each provider’s type (e.g. medical doctor, physician assistant, and technician), age, gender, year of medical school graduation, treatment assignment, and encounter location (i.e. Hospital non-ED, ED, or Outpatient). Finally, patient-level data from Aurora’s Epic electronic medical record system provided information on the age and gender of the patient as of February 6, 2018.

## III.2 Variable Definitions

High-cost imaging is comprised of CT, MRI, PET and nuclear medicine. The most common types of high-cost imaging orders are CT scans and MRIs. Unless otherwise-specified, all of our scan order measures refer to high-cost scans.

Our primary outcome is the number of targeted scans ordered during our study period (December 15, 2016 through December 15, 2017). A targeted scan is a scan-indication pair for which the BPA would be shown throughout our study period; these are scans with a score of less than 7 with a strictly higher scoring alternative. We define targeted scans symmetrically for both the treatment and control providers, even though the BPA is only shown to the treatment providers.

We examine a number of secondary outcomes. We partition targeted scans into those that are scored 1-3 (red orders) and those that score 4-6 (yellow orders); we also separately examine the two most common types of targeted scans: targeted CT scans, and targeted MRI scans. Finally, we examine the total number of high-cost scans (both targeted and non-targeted), as well the number of low-cost scans. Low cost-scans – including ultrasound, x-ray, mammograms, fluoroscopy - are potentially substitutes for low-scoring high-cost scans.

All of our scans measures refer to scans that are ordered by providers in our study population, regardless of whether they are actually performed. This is because we are interested in the provider’s decision at the time of ordering. Orders may not be performed if, for example, the patient does not show up to get the ordered scan or providers update their orders based on new medical information obtained at a later date.

## III. 3 Summary statistics.

### Distribution of ordering behavior

Appendix Table 1 shows distributional statistics on scan orders of control providers over the study period. Ordering is highly skewed. The bottom quartile of providers order 2 or fewer high-cost images, while the top quartile ordered 125 or more. More than one-quarter of providers ordered zero targeted scans, while the 75^th^ percentile provider ordered 18 or more targeted scans.

Appendix Table 2 shows the distribution of scan *types* ordered by control providers during our study period. About 90 percent of high cost scans are MR or CT, with CT being about twice as common (60%) as MRI (30 %). Another 8 percent of high-cost scans are nuclear medicine, and about 2 percent are PET scans. Low-cost scans are about four times more commonly ordered than high-cost scans. The shares of low-cost imaging are: ultrasound (26.1%), x-ray (50.8%), mammogram (15.9%), fluoroscopy (5.0%), and bone densitometry (2.2%), which we include for completeness.

Appendix Table 3 shows the 20 most frequently requested indication-scan type pairings for high cost scans ordered by controls during the study period. Appendix Table 4 shows the 20 most frequently requested indication-image parings for targeted scans.

### Unscored Scans

As noted in Section II, not all scans are scored. Appendix Table 5 shows information on unscored scans. Panel A shows that about 22% of high cost scans ordered by controls during our study period were unscored. It also shows that while the vast majority of scored scans had scores assigned directly by the CDS, in about 4 percent of cases we imputed scores to handle issues such as server downtime. For these scans, we combined indication and imaging orders from the EMR with the rule set supplied by NDSC; this allowed us to map imaging-order requests to ACR Select scores, and to scores for the alternative scans for that indication. Below, we refer to such cases as ones with “scores imputed”.

Panel B of Appendix Table 5 shows the set of reasons for a scan to be unscored. The three largest categories are (1) Medicalis scans – four specific scans (CT angiogram coronary arteries, NM myocardial perfusion rest or stress multi, NM myocardial perfusion rest or stress single, and NM PET myocardial perfusion) - which Aurora chose not to have ACR Select score since they were already subject to a different CDS system - (2) some oncology indications - which ACR Select doesn’t score - and (3) procedures that were not in the ACR Select rule set .

# IV. Estimating Equation

Our analyses compare outcomes for ordering providers who were randomized to the treatment group to those who were randomized to the control group. Our baseline specification is the following OLS regression at the provider level:

$Y_{i}=\beta_{0}+\beta_{1}{1(\text{Treatment})}_{i}+\beta_{2}X_{i}+\varepsilon_{i}$ (1)

where ${1(\text{Treatment})}_{i}$ is an indicator variable equal to one if the ordering provider *i* was randomized to the treatment group and zero if the ordering provider was randomized to the control group; $X_{i}$ is a vector of control variables. We report heteroskedasticity-robust standard errors.

$\beta_{1}$ is the parameter of interest; it measures the impact of the ordering provider being randomized into the treatment group on the outcome Y.

Given our simple randomized design, we do not need to control for any covariates $X_{i}$ to produce an unbiased estimate of $\beta_{1}$. However, because our historical power calculations suggested that it substantially increased statistical power, our baseline specification includes as $X_{i}$a control for the lag of the dependent variable - measured during the quiet period before the BPA could be shown to providers.^33,34^

# V. Additional Results

## V.1 Sensitivity analysis

We explored the robustness of our main results (Table 2 in the text). Appendix Table 6 shows results for our primary outcome – targeted scans. The first row replicates the baseline specification from Table 2 (described above). The next two rows show results without this control, or with additional controls, respectively. Not controlling for the lagged dependent variable substantially reduces the precision of our estimate (i.e. increases the width of the 95% confidence interval), while expanding the set of control variables does not appear to have much effect. This is what we expected based on our power calculations using historical data.^33,34^

The next two rows show results from analysis using nonlinear models instead of OLS. Given that our outcome is a count variable, we report results from a negative binomial model and a quasi-maximum likelihood poisson model.^35^ In both models the results are quantitatively similar to the baseline OLS results.

The following two rows explore the sensitivity of our analysis to alternative ways of measuring the primary outcome. In the first of these two, we limit our outcome measure to targeted scans that are actually performed on patients by the end of our study period; about 22% of orders do not result in a scan performed. The penultimate row defines our primary outcome based on the CDS logic that was in place for the first few months of the study. Our main findings are not sensitive to the use of either alternative measure.

In the next row, we explore robustness to the exclusion of the 402 providers (194 treatment and 208 controls) who left Aurora at some point before the end of our study period. This exit rate is balanced across treatment and control; (t-test of balance has p-value=0.46). Some of these providers left during the quiet period; however the 144 control providers and 133 treatment providers who left Aurora during our study period (rather than before it) are also balanced across treatment and control (t-test of balance has p-value of 0.49). We do not know the exact date of departure, which is why they are included in our main analysis. However the final row shows that effects are similar if we limit to the subsample who was at Aurora throughout the quiet and study period.

In the last two rows, we considered the potential impact of taking into account that providers share practice locations. Specifically, using encounter-level data, we identified each provider’s primary location (hospital or clinic where they had the largest number of encounters over the trial period). We then tested the effects of hierarchical modeling by augmenting the main specifications to estimate the confidence intervals clustering the standard errors at this primary-location level, which accounts for correlation across providers within location (sometimes referred to as Eicker-Huber-White heteroscedasticity-robust standard errors with clustering) and estimated using the cluster command in STATA version 13.^36,37^ We also estimated models that included provider primary-location fixed effects (indicator variables for each location) and again clustered the standard errors at the level of the provider’s primary location. In both analyses, we found very similar confidence intervals and corresponding p-values when taking this hierarchical structure into account.

## V.2 Strategic Avoidance of CDS

### Handoffs to providers not subject to CDS

Providers may try to avoid the CDS, either to avoid the time cost associated with it and/or to protect themselves against repercussions from oversight based on CDS recommendations. Such avoidance can limit the effectiveness of the CDS.

In Appendix Table 7, we empirically investigate the two main ways that providers could avoid the CDS system.

As noted in Section II above, ordering providers decide whether and what image to order, but they may not be the person who enters the order (the “user”). When these differ we refer to the order as a “handoff”. Handoffs by one of our study providers could be to another study provider, or to providers outside of the study population – this is most commonly a nurse, but may also be a technician.

It is the status of the person entering the order (the “user”) that determines whether or not it is subject to the CDS. Therefore, one way to avoid the CDS is for the ordering provider to hand off the order entry to a user provider who is not subject to the CDS. This would be interesting both as a potential signal of provider antipathy to the CDS, and as a way the impact of the CDS could be weakened. In our study context it could also mean handing off the order entry to a control provider; this would be a threat to the validity of our empirical design.

Panel A of Table 7 investigates handoffs to providers not subject to the CDS. It shows that, during our study period, almost one-quarter of orders by control providers were handed off – i.e. entered by user providers who were different from the ordering provider. The vast majority of these handoffs were to providers outside of our study population – primarily nurses; about 1 percent of handoffs were to other control providers. There is no evidence that the treatment affected the share of orders handed off, either overall or to control providers in particular. In other words, we detect no evidence of strategic avoidance of the CDS through handoffs.

However, as discussed in the main text, almost one-quarter of orders in our study that are supposed to be subject to the CDS are placed by providers who are not subject to the CDS. To account for the fact that handoffs preclude treatment providers from interacting with CDS, we may want to rescale our estimates of the impact of CDS to reflect that treatment providers are not exposed to CDS for all of their orders. In other words, our point estimate that CDS reduced the number of targeted scans by 1.1 represents a 6% decline relative to the 17 targeted scans ordered on average by control providers; but another interpretation is that on average only about three-quarters of targeted scan orders are likely to be placed by user providers covered by CDS, suggesting that the 1.1 decline represents an 8 percent decline relative to the approximately 12.75 targeted scans that were subject to the CDS.

### Reduced patient volume

Panel B explores a second way providers could avoid the CDS which is to see fewer patients. This would also be a signal of provider or patient antipathy to the CDS, and would pose a threat to the validity of our empirical design if patients were substituted from treatment providers to control providers. Panel B shows no evidence of CDS affecting the number of patient encounters.

### Indication changes

Yet another way to avoid the CDS is for providers to change the indication for the scan they are ordering, so that the order receives a higher score. This would nominally increase the appropriateness of the order without any real change in ordering behavior. One might expect that any such “indication gaming” would increase over time as providers learn how to change the indications to avoid the CDS. Our finding that the impact of CDS does not decrease over the time period of our study does not support this hypothesis. In addition, we implemented a balance test for whether the distribution of indications on scan orders differs between the treatment and control group; specifically, for the top 30 indications in the quiet period (accounting for about half of quiet period scan orders), we estimated an OLS regression at the scan level of a binary variable for treatment status on indication indicators. This is a valid test of changes in indications only if the number of high-cost imaging orders did not change due to CDS, since a change in ordering could naturally lead to a change in indication frequency among the remaining orders. An F-test of equality of the coefficients on the indication indicators is unable to reject the null of the same distribution of indications across treatment and control scan orders (p=0.56). In addition, for the full set of indications ordered, we conducted a chi-squared test of equality of the distributions for treatment and control orders in the study period relative to the quiet period, and again fail to reject the null of equality (p=0.72).

Choosing such unscored indications represents another pathway to avoid the CDS. Most unscored scans, shown in Appendix Table 5 are unscored because of the indication choice; this includes free-text indications entered by a technician or clerical worker—individuals who do not select a structured indication despite the mandate that other providers such as physicians and nurses do so—and structured indications that do not produce a score (such as oncology indications). Appendix Table 5 shows that the CDS intervention had no impact on the rate of these unscored scans.

# **Figure S1: Project Timeline**


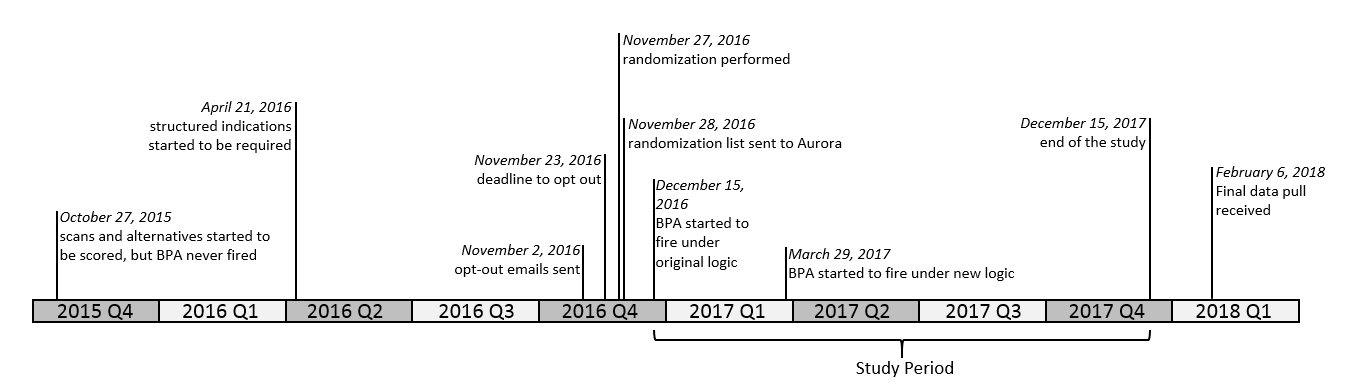


# **Table S1: Distributional Statistics on Orders Placed by Control Providers**

# **Table S2: Types of Imaging Orders Placed by Control Providers**

**Table S3: Top 20 Most Frequent Indication-Imaging Pairs Ordered By Control Providers**

**Table S4: Top 20 Most Frequent Targeted Indication-Imaging Pairs Ordered By Control Providers**

# **Table S5: Impact of CDS on Scores of Imaging Orders**

# **Table S6: Sensitivity analysis for Impact of CDS on Primary Outcome (Targeted Scans)**

# **Table S7: Analysis of Potential CDS Avoidance**

#

# VI. References

1. Bates DW, Leape LL, Cullen DJ, et al. Effect of Computerized Physician Order Entry and a Team Intervention on Prevention of Serious Medication Errors. *JAMA*. 1998;280(15):1311–1316.

2. Gurwitz JH, Field TS, Rochon P, et al. Effect of Computerized Provider Order Entry with Clinical Decision Support on Adverse Drug Events in the Long-Term Care Setting. *J Am Geriatr Soc*. 2008;56(12):2225–2233.

3. Schnipper JL, Hamann C, Ndumele CD, et al. Effect of an Electronic Medication Reconciliation Application and Process Redesign on Potential Adverse Drug Events: a Cluster-Randomized Trial. *Arch Intern Med*. 2009;169(8):771–780.

4. Raschke RA, Gollihare B, Wunderlich TA, et al. A Computer Alert System to Prevent Injury from Adverse Drug Events: Development and Evaluation in a Community Teaching Hospital. *JAMA*. 1998;280(15):1317–1320.

5. Bates DW, Kuperman GJ, Rittenberg E, et al. A Randomized Trial of a Computer-Based Intervention to Reduce Utilization of Redundant Laboratory Tests. *Am J Med*. 1999;106(2):144–150.

6. Bates DW, Kuperman GJ, Rittenberg E, et al. Reminders for Redundant Tests: Results of a Randomized Controlled Trial. In: *Proceedings of the Annual Symposium on Computer Application in Medical Care*. American Medical Informatics Association; 1995:935. http://www.ncbi.nlm.nih.gov/pmc/articles/PMC2579250/. Accessed January 19, 2015.

7. Thomas RE, Croal BL, Ramsay C, Eccles M, Grimshaw J. Effect of Enhanced Feedback and Brief Educational Reminder Messages on Laboratory Test Requesting in Primary Care: a Cluster Randomised Trial. *The Lancet*. 2006;367(9527):1990–1996.

8. Bell LM, Grundmeier R, Localio R, et al. Electronic Health Record–Based Decision Support to Improve Asthma Care: a Cluster-Randomized Trial. *Pediatrics*. 2010;125(4):e770–e777.

9. Bourgeois FC, Linder J, Johnson SA, Co J, Fiskio J, Ferris TG. Impact of a Computerized Template on Antibiotic Prescribing for Acute Respiratory Infections in Children and Adolescents. *Clin Pediatr (Phila)*. 2010;39(10):976-983.

10. Christakis DA, Zimmerman FJ, Wright JA, Garrison MM, Rivara FP, Davis RL. A Randomized Controlled Trial of Point-of-Care Evidence to Improve the Antibiotic Prescribing Practices for Otitis Media in Children. *Pediatrics*. 2001;107(2):e15.

11. Davis RL, Wright J, Chalmers F, et al. A Cluster Randomized Clinical Trial to Improve Prescribing Patterns in Ambulatory Pediatrics. *PLoS Clin Trials*. 2007;2(5):e25.

12. Field TS, Rochon P, Lee M, Gavendo L, Baril JL, Gurwitz JH. Computerized Clinical Decision Support During Medication Ordering for Long-term Care Residents with Renal Insufficiency. *J Am Med Inform Assoc*. 2009;16(4):480-485. doi:10.1197/jamia.M2981

13. Terrell KM, Perkins AJ, Dexter PR, Hui SL, Callahan CM, Miller DK. Computerized Decision Support to Reduce Potentially Inappropriate Prescribing to Older Emergency Department Patients: a Randomized Controlled Trial. *J Am Geriatr Soc*. 2009;57(8):1388-1394. doi:10.1111/j.1532-5415.2009.02352.x

14. van Wyk JT, van Wijk MAM, Sturkenboom MCJM, Mosseveld M, Moorman PW, van der Lei J. Electronic Alerts Versus On-Demand Decision Support to Improve Dyslipidemia Treatment: A Cluster Randomized Controlled Trial. *Circulation*. 2008;117(3):371-378. doi:10.1161/CIRCULATIONAHA.107.697201

15. Bright TJ, Wong A, Dhurjati R, et al. Effect of Clinical Decision-Support Systems: a Systematic Review. *Ann Intern Med*. 2012;157(1):29-43. doi:10.7326/0003-4819-157-1-201207030-00450

16. Georgiou A, Prgomet M, Markewycz A, Adams E, Westbrook JI. The Impact of Computerized Provider Order Entry Systems on Medical-Imaging Services: a Systematic Review. *J Am Med Inform Assoc*. 2011:335–340.

17. Main C, Moxham T, Wyatt JC, Kay J, Anderson R, Stein K. Computerised Decision Support Systems in Order Communication for Diagnostic, Screening or Monitoring Test Ordering: Systematic Reviews of the Effects and Cost-Effectiveness of Systems. 2010:1-227.

18. Tajmir S, Raja A, Ip I, et al. Impact of Clinical Decision Support on Radiography for Acute Ankle Injuries: a Randomized Trial. *West J Emerg Med*. 2017;18(3):487-495.

19. Bates DW, Kuperman GJ, Jha A, et al. Does the Computerized Display of Charges Affect Inpatient Ancillary Test Utilization? *Arch Intern Med*. 1997;157(21):2501–2508.

20. Bunt CW, Burke HB, Towbin AJ, et al. Point-of-Care Estimated Radiation Exposure and Imaging Guidelines Can Reduce Pediatric Radiation Burden. *J Am Board Fam Med JABFM*. 2015;28(3):343-350. doi:10.3122/jabfm.2015.03.140251

21. Gimbel RW, Fontelo P, Stephens MB, et al. Radiation Exposure and Cost Influence Physician Medical Image Decision Making: A Randomized Controlled Trial. *Med Care*. 2013;51(7):628-632. doi:10.1097/MLR.0b013e3182928fd5

22. Gimbel RW, Pirrallo RG, Lowe SC, et al. Effect of clinical decision rules, patient cost and malpractice information on clinician brain CT image ordering: a randomized controlled trial. *BMC Medical Informatics and Decision Making* 2018;18(1):20.

23. Rosenthal DI, Weilburg JB, Schultz T, et al. Radiology Order Entry With Decision Support: Initial Clinical Experience. *J Am Coll Radiol*. 2006;3(10):799-806. doi:10.1016/j.jacr.2006.05.006

24. Lin FY, Dunning AM, Narula J, et al. Impact of an Automated Multimodality Point-of-Order Decision Support Tool on Rates of Appropriate Testing and Clinical Decision Making for Individuals with Suspected Coronary Artery Disease: a Prospective Multicenter Study. *J Am Coll Cardiol*. 2013;62(4):308–316.

25. Solberg LI, Wei F, Butler JC, Palattao KJ, Vinz CA, Marshall MA. Effects of electronic decision support on high-tech diagnostic imaging orders and patients. *Am J Manag Care*. 2010;16(2):102–106.

26. Sistrom CL, Dang PA, Weilburg JB, Dreyer KJ, Rosenthal DI, Thrall JH. Effect of Computerized Order Entry with Integrated Decision Support on the Growth of Outpatient Procedure Volumes: Seven-Year Time Series Analysis. *Radiology*. 2009;251(1):147–155.

27. Vartanians VM, Sistrom CL, Weilburg JB, Rosenthal DI, Thrall JH. Increasing the Appropriateness of Outpatient Imaging: Effects of a Barrier to Ordering Low-Yield Examinations. *Radiology*. 2010;255(3):842–849.

28. Huber TC, Krishnaraj A, Patrie J, Gaskin CM. Impact of a Commercially Available Clinical Decision Support Program on Provider Ordering Habits. *Journal of the American College of Radiology* 2018;15(7):951–7.

29. Bookman K, West D, Ginde A, et al. Embedded Clinical Decision Support in Electronic Health Record Decreases Use of High-cost Imaging in the Emergency Department: EmbED study. *Acad Emerg Med* 2017;24(7):839–45.

30. Timbie JW, Hussey PS, Burgette L, et al. Medicare Imaging Demonstration Evaluation Report for the Report to Congress. *St Monica CA Rand Corp*. 2014.

31. Bode FR, Cascade PN. The American College of Radiology Appropriateness Criteria : Will They be Useful for Us? *Chest*. 1996;110(4):869-871. doi:10.1378/chest.110.4.869

32. American College of Radiology. ACR Appropriateness Criteria: Overview. February 2015. https://www.acr.org/Clinical-Resources/ACR-Appropriateness-Criteria/Overview.

33. Doyle J, Abraham S, Feeney L, Reimer S, Finkelstein A. Clinical Decision Support (CDS) for Radiology Imaging: a Randomized Control Trial. December 2016. https://www.socialscienceregistry.org/trials/1846.

34. Doyle J, Abraham S, Feeney L, Reimer S, Finkelstein A. Clinical Decision Support (CDS) for Radiology Imaging. December 2016. https://clinicaltrials.gov/ct2/show/record/NCT02996045.

35. Cameron AC, Trivedi PK. *Regression Analysis of Count Data*. Vol 53. Cambridge university press; 2013.

36. White H. *Asymptotic theory for econometricians.* Rev. ed. San Diego: Academic Press; 2001.

37. Liang K-Y, Zeger SL. Longitudinal data analysis using generalized linear models. *Biometrika* 1986;73(1):13–22.
