# Supplementary material for: Clinical decision support for high-cost imaging: A randomized clinical trial
Source: PLoS One. 2019 Mar 15;14(3):e0213373. doi: 10.1371/journal.pone.0213373 (PMC6419998; doi:10.1371/journal.pone.0213373)
Supplement: S1 Trial Protocol — (DOCX) [file pone.0213373.s003.docx]

Below is the trial protocol approved by the Aurora IRB prior to the beginning of the trial. For primary and secondary outcomes, please refer to the trial registries:

<https://clinicaltrials.gov/ct2/show/NCT02996045>

<https://www.socialscienceregistry.org/trials/1846>

**Proposal Title: The Impact of Clinical Decision Support on Advanced Imaging Ordering: A Randomized Control Trial**

**PI: Sarah Reimer, MD**

**Title: Diagnostic Radiologist, Medical Director of Population Health and Risk**

**Address: 14290 Green Bay Road, Mequon, WI 53097**

**Phone: 414-265-9243**

**Email: sarah.reimer@aurora.org**

**SPONSOR**

MIT is the author of the protocol and the main grant awardee. Aurora Health Care is the potential sub-awardee of a grant from the Laura and John Arnold Foundation (LJAF).

**Version Date: July 13, 2016 (replaces** May 19, 2016)

**BACKGROUND AND SIGNIFICANCE**

In 2008, the Congressional Budget Office identified inappropriate use of health services as a major contributor to health care spending inefficiency [1]. In 2013, the Medicare Payment Advisory Commission (MedPAC) identified the inappropriate use of diagnostic imaging as a large part of the problem [2]. It is estimated that one third of imaging procedures are not warranted, costing between $3 billion to $10 billion annually while there is no clear guidance on clinical effectiveness (i.e., more tests do not result in better health outcomes; on the contrary they might even lead to unnecessary exposure to radiation and subsequent testing) [3]. The issue holds for both the inpatient and ambulatory setting. In a study conducted in the ambulatory setting rates of as high as 62% (for CT of the head) of inappropriate use of imaging were found. Moreover, the study also demonstrated higher rates of negative results when tests were deemed inappropriate (47%) versus those that were deemed appropriate (20%) [4].

**Clinical Decision Support (CDS)**

Health information technology has the potential to remind providers about appropriate health utilization [1]. Many health insurers are now using 'radiology benefit management' programs to reimburse imaging use by evaluating them against pre-specified appropriateness criteria [3]. Another option that is being utilized is Clinical Decision Support (CDS) software. CDS generally refers to the use of software systems to improve clinical decision-making, and involves matching patient characteristics to evidence-based guidelines to provide clinicians with clinically relevant information at the time of order entry.

In particular, we intend to study the use of a tool called ACR Select[7]. ACR Select is the electronic version of the American College of Radiology's Appropriateness Criteria at point of order and is considered the industry standard. When ordering a scan through Epic’s order entry system, providers choose a clinical indication and a particular scan (e.g. the indication is a headache and the scan is a CT Scan of the Head). The patient’s age and gender, along with the indication and scan are then evaluated against the ACR guidelines. A best-practice alert (BPA) can then be generated—a pop-up screen that describes the results. Scores range from 1-9, with 1 being the least appropriate and 9 the most. In accordance with the Appropriateness Criteria, the numeric scores are grouped into three categories for the purpose of providing recommendations to decision-makers: 1-3 is deemed “usually not appropriate”, 4-6 “may be appropriate”, and 7-9 “usually appropriate.” The Appropriateness Criteria represent a national standard and were developed by an interdisciplinary panel of experts in the field of radiology and medicine to determine appropriate/inappropriate use of diagnostic imaging.

A typical BPA screen looks like this (We are in the process of editing this to include a note on the top that says “Unless further action is taken in the checkboxes below, CT HEAD W CONTRAST will be removed from orders":


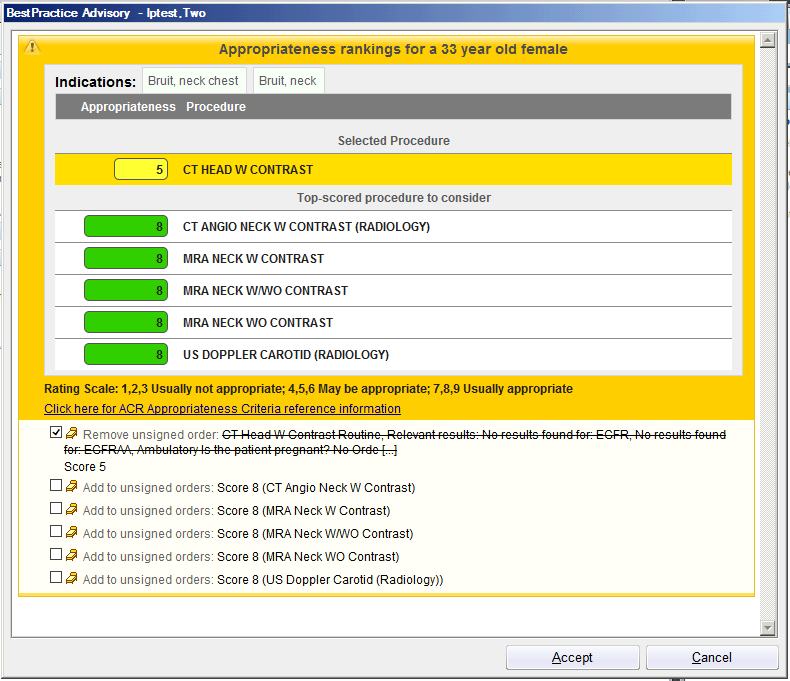


Results from observational pre-post studies suggest that the introduction of such clinical decision support systems in outpatient radiology may reduce high-cost scanning by about 20% overall, and reduce the rate of “inappropriate” scanning by 20-70%. [5, 6]. However, there has never been a randomized trial of the impact of CDS for radiology orders, which leaves considerable uncertainty about the impact of CDS for radiology. For example, it is possible that the estimated impact of CDS in pre-post studies is contaminated by other initiatives or changes going on in the healthcare system at the same time that CDS was introduced. The researchers involved in this study are currently implementing a randomized trial in another setting, and hope to combine evidence from multiple settings to generate a body of evidence around CDS.

**CDS at Aurora**

Aurora Health Care implemented ACR select in October 2015. It is currently running “in the background” with no best-practice alerts being shown to providers. Best practice alerts will begin to be shown to providers in the near future in part because of new federal regulations. It is important to note that providers maintain their authority over imaging and treatment plans. The CDS intervention (Best Practice Alert) is non-obtrusive and can be easily overridden by the provider.

The Protecting Access to Medicare Act (PAMA) of 2014 [9] stipulates that provider-led entity developed CDS (such as ACR Select) must be consulted at the time of order entry for advanced diagnostic imaging performed on ambulatory Medicare patients. (Originally, the law specified January 1, 2017. The most recent regulatory commentary states that it will not be mandated by January 1, 2017 but does not specify when it will be mandated.) The law defines the ambulatory setting as inclusive of the provider office building or clinic, outpatient imaging performed in a hospital, the Emergency department, and the ambulatory surgery center. If proof of CDS consultation at order entry and a measure of how well the choice of imaging aligns with the appropriateness criteria are not attached to each Medicare claim, both the technical and professional reimbursement for the exam will be forfeit. Nearly all of Aurora Health Care's AboutHealth partners have implemented or are in the process of implementing CDS for advanced imaging across their systems, both inpatient and outpatient. To comply with the law and to stand with our partners in providing the most appropriate imaging care, Aurora Health Care has also done this.

While all providers are now entering a clinical indication for exam, the appropriateness is being scored in the background and stored without being shown to providers. This step, which is commonly taken by systems implementing CDS, allows measurement of baseline imaging utilization and appropriateness. We anticipate that when the mandate takes effect, the appropriateness scores and associated clinical decision support will be shown to all ordering providers at the point of order entry.

Our study will allow Aurora to understand the value of the CDS tool for providers and patients and also helps roll out the system in a systematic way to anticipate effects of complying with the law. As CMS has expressed informal interest in the results of this study, it may impact healthcare delivery nationally. This proposal has been outlined to and received support from Aurora Health Care's senior leadership.

**OBJECTIVE**

The overall goal of this research is to evaluate the impact of a CDS system on the appropriateness of advanced imaging ordering.

We aim to determine whether decision support will:

1. Lower the rate of low utility scans (ACR scored 1-3).

2. Lower the rate of high-costs scans.

3. Affect downstream utilization. Decision support could, in theory, increase, decrease, or leave these costs unchanged.

**ENDOINTS**

Primary endpoints are:

- Ordering rate of scans of low utility (ACR score 1-3). This will be recorded as the number of inappropriate scans and the number of inappropriate scans per visit to the provider

- Provider diagnostic imaging rates: # of CT, # of MRI and # of total CT and MRI scans, as well as these measures per visit to the provider

Secondary endpoints are:

- Indication of change or cancellation of a scan

- Indicators of subsequent healthcare utilization/costs, such as:

• Subsequent scans

• ED visits

• Clinic visits

• Inpatient admission rate

• Number of inpatient admissions

• Procedures

**STUDY POPULATION AND SIZE**

**Providers:** After analyzing the data from the historical data pull, we will study all providers with the ability to order advanced imaging. The exact number will be determined after analyzing the data, but we expect it to be on the order of 5000 providers. We will exclude CDS data from those providers who opt out. The categories of providers who are authorized to order advanced imaging include MD, DO, physician assistant, and nurse practitioner. Email address lists of providers in these provider categories will be obtained from Aurora and its affiliates. The opt-out email will be sent to these email addresses. This email includes a paragraph that providers are advised to read to their patients, in order notify them of the study.

**Patients:** Visits from all patients who are seen by providers in the study will be included and their outcomes analyzed. CDS may result in better-informed providers such that they are more likely to follow the American College of Radiology guidelines. This includes deciding not to scan. As a result, it is necessary to study outcomes for all patient visits, exclusive of behavioral health and excluding minors (age less than 18 years).

**EXPERIMENTAL DESIGN AND METHODS**

We propose a prospective, randomized controlled trial to measure the impact of CDS. Providers will be randomized into two arms: a treatment group that will receive feedback from the CDS system and a control group that does not.

- Aurora will send MIT a list of providers (encrypted IDs).  This will be for all providers who ordered an exam over the past 2 years who did not opt out of the study.
- MIT will randomize and create an indicator for treatment/control

(=0 or 1)

- MIT will send the encrypted IDs and their treatment indicator to Aurora
- Aurora will attach the treatment indicator to actual provider IDs
- The actual provider IDs and treatment indicator will then be sent by Aurora to NDSC*
- The order-entry system sends the order details, including the provider ID, to NDSC.  NDSC will write a script to display the best practice alerts only to those provider IDs in the treatment group.

Aurora will create and maintain the actual ID - encrypted ID crosswalk.

This will not be provided to MIT or NDSC.

*NDSC is the national decision support company. They provide the ACR Select

software. The provider ID will be sent to NDSC as part of the implementation

of ACR Select – and is not a research piece.

The CDS consists of several (bundled) components:

1. **Require selection of indication.** In order to properly score as many scans as possible, the order-entry software has been configured to require ordering providers to select a discrete indication from an existing list. This has been and will continue to be required of all providers: both treatment and control.
2. **Optional textbox to clarify indication selection**. There is an *optional* free-text box below the indication that allows providers to provide relevant additional clinical information for radiologists. This is available to all providers: both treatment and control.

The remaining features will be turned on only for providers in the treatment group and for providers who have opted out of the study. The below specifics are a business decision and a starting point for this project, independent of the research, and as such, are subject to modification.

1. **Display CDS at provider sign-off .** The CDS displays as a pop-up screen which appears when providers attempt to sign off on a scan. Dr. Reimer will work with informatics physician leaders to determine a firing logic which minimizes provider alert fatigue.
2. **Only show appropriateness score.** The decision support tool will only show the appropriateness score, not costs or radiation exposure levels – though costs and radiation exposure levels are options on the CDS software.
3. **Only display alternative scans with a score of 7-9.** Only alternative scans with scores greater than 6 will be displayed in the CDS window. Only the highest rated scan alternative(s) will appear as “suggested” alternatives, while other alternative scans rated 7-9 will appear in the CDS window in order of appropriateness (*i.e.* first is the highest rated scan, etc.). This is an attempt to make it easiest to order the “most appropriate” alternative scan.
4. **Default to cancel scans.** Any time the CDS pop-up appears, a checkbox to remove the selected scan will be checked by default. This default can be over-ridden by the ordering provider by de-selecting the checkbox.^[[1]](#footnote-1)^
5. **Provide relevant ACR documentation.** A link to ACR documentation relevant to the indication category will be provided in the pop-up window.

**Informed Consent**

We are requesting an alteration of informed consent and a waiver of documentation of informed consent for the provider. We are requesting a waiver of Informed consent for the patient.

The following sections describe the justification that this research meets the definition of “minimal risk” as defined in the Common Rule.

**Minimal Risk Justification**

**Definition of Minimal risk:** “the probability and magnitude of harm or discomfort anticipated in the research are not greater in and of themselves than those ordinarily encountered in daily life or during the performance of routine physical or psychological examinations or tests”

**Provider + RCT + ACR Select**

Clinical decision support is commonly encountered in providers’ daily life on a national, regional, and local level. Providers experience ACR Select in daily life at about 80 institutions across the nation. Regionally, ACR Select either has been or is being implemented by most members of AboutHealth, the 8-member alliance of health systems in Wisconsin, of which Aurora is a founding member. Currently, providers in Aurora routinely encounter best practice alerts of many sorts in their normal use of the EMR (Epic). Absent this study, all of our providers would be seeing ACR Select decision support well ahead of the required date set by CMS.

The subjects may receive care that is more likely to follow the American College of Radiology guidelines. As the guidelines are considered best practice, we do not anticipate any adverse effect for these patients.

**Provider + RCT + No ACR Select Feedback**

This is current practice, and the risk of not using ACR Select is not greater than those ordinarily encountered in daily life.

**Patients of the providers (both with and without ACR Select)**

Insurers currently use a variant of appropriateness criteria for radiology benefit management, and the benefit or harm to patients is unproven and not measured. Aurora does not require preauthorization of patients for advanced imaging in the Aurora Accountable Care Network, which includes employees and others. So, we are currently applying CDS variably across our patient populations without any informed consent, and without understanding how this impacts patient care or costs. This current practice is not research, as we are not collecting/measuring data in a manner that constitutes to generalizable knowledge. However, this is something that patients encounter in daily life as a patient.

As a patient of a provider receiving ACR Select – a patient routinely sees providers who have different levels of technological access, and this is part of their everyday life as a patient.

The control group receives usual care as they are currently receiving it, therefore we do not anticipate any adverse effect of participating in the research for these patients.

**The waiver or alteration will not adversely affect the rights and welfare of the subjects**

**Providers as subjects:** The alteration of informed consent will not adversely affect the rights and welfare of the providers.

The decision to implement this clinical decision support rests with the organization, not with the individual provider. Although the providers now do not see the ACR Select decision support, they will see it ahead of the law’s requirement, regardless of whether or not this research is implemented.

Trial participation status will not impact employment, social status, or reimbursement to the employer.

Providers will be told about this study, and have the opportunity to opt out of having their CDS data analyzed. If they opt out, they will see the ACR Select decision support, as this will be the default for Aurora. Providers who opt out of the study prior to its start will not be randomized.

**Patients as subjects:** Patients’ rights and welfare will not be adversely affected by this waiver of informed consent. Their providers will continue to direct their advanced diagnostic imaging care based upon their training, experience, and ongoing review of the literature, upon which the appropriateness criteria are based.

**The research could not practicably be carried out without the waiver or alteration**

**Providers:** This research could not be practicably carried out without the alteration, which is very close to a waiver of Informed consent.

There is no practicable way to carry out this research without such an alteration. Anything less than almost full participation would skew the data. Opt-in programs typically have a low participation rate. For example, the simple difference between an opt-in (explicit consent) and an opt-out (presumed consent) process can push organ donation rates from, say, 4.25 per cent (in Denmark) to 99.98 per cent (in Austria). A low participation rate would severely limit the availability of data.

An e-mail notification that is brief, concise, and provides an opt out option and an opportunity to ask questions is a respectful, responsible way to inform the providers of this research, and have this research move forward. This research meets the definition of minimal risk, and does not adversely affect the rights and welfare of the subjects.

**Patients:** This research could not be practicably carried out without a waiver of Informed consent

There is no practicable way to carry out this research without a waiver. This study will be implemented at the provider level. This leads an informed consent process for patients to be impracticable for the following reasons:

1. We believe a part of the effect of the decision support may be from providers learning over time which types of scans are more or less appropriate in different clinical scenarios. It is impracticable for patients to decide not to be part of such a study because the providers are learning from their experience with other patients.
2. For this learning to occur as it would outside of a trial or research perspective, providers must have the opportunity to view the CDS alerts for each patient – not just a subset who consent. The characteristics of patients who consent to the study are likely to be systematically different from those who do not consent. This would bias the results of the research if consenters are more (or less) likely to have a condition for which a provider would order a more (or less) appropriate scan.
3. The consent process is likely to cause increased administrative burden, and impede or delay the normal course of care, as the patient reviews the consent form.
4. It is technologically infeasible to de-activate the decision support software for patients who do not consent.
5. All patients at Aurora see providers who could place an order, and consenting of all patients would be a monumental task and one that would result in the study not being performed.

Information on utilization and underlying health characteristics are essential for the measurement of the outcomes of this study: scan ordering patterns and downstream health utilization. Only de-identified information will be shared with MIT. This de-identified information will be coded, and Aurora will maintain the code (explained in Data Collection Section).

This research meets the definition of minimal risk and does not adversely affect patients’ rights and welfare.

**Whenever appropriate, the subjects will be provided with additional pertinent information after participation**

**Providers:** There will be no additional pertinent information to provide to providers after participation. The results may impact CMS decision regarding use/implementation. If a publication results, it will be available to providers throughout Aurora by means of ALIS.

**Patients:** There will be no pertinent information to provide to the patients as a result of the study.

**Waiver/Alteration of Informed Consent (45 CFR 46)**

This study meets the definition of Minimal Risk, and as such we are asking for an Alteration of Informed Consent and Waiver of documentation of informed consent for the provider. This alteration is in the form of an opt out email notification.

**Provider notification (Alteration of Informed Consent, opt out)**

The categories of providers who are authorized to order advanced imaging include MD, DO, physician assistant, and nurse practitioner. Email address lists of providers in these provider categories will be obtained from Aurora and its affiliates. The opt-out email will be sent to these email addresses. This email includes a paragraph that providers are advised to read to their patients, in order notify them of the study.

Dr. Sarah Reimer and her staff will coordinate the notification of providers about the study. This will be in the form of an email provided as an addendum to this proposal. This coordination will entail responding to provider questions and keeping track of any provider who opts out of the study. Opting out of the study will mean that the data when that provider is the ordering provider will not be analyzed. Providers who opt out will receive the CDS tool, as it has already been adopted by Aurora and will be universally adopted in the near future. To clarify from the previous submission, these providers are not randomized—they are not part of the study.

The providers will be given several weeks to ask questions and / or opt out of the study.  It will take time for the research team to respond to questions and assemble the opt-out list.  Randomization will occur after the opt-out list has been assembled and given to Data Warehouse and Research Analytics.

**DATA COLLECTION**

The Data pull will be at the encounter-scan level. If there is no scan or one scan associated with an encounter, then there will be one record for that encounter. If there is more than one scan associated with an encounter, then there will be multiple records for that encounter (one for each scan). “Scan” means any study (including x-ray, ultrasound, CT, MR, PET, etc).

Patient and provider identifiers are needed at Aurora Health Care for the purpose of linking data elements across data sources. The data extraction process will be performed through each database separately in secured areas for patient data at Aurora Health Care.

The PI and team will combine these data and create a De-identified Data Set that will be shared with researchers from MIT. The data element list includes data from Epic, TSI, MSO, Strata, and NDSC. The data will include proxy unique identifiers for each patient and ordering provider to allow for tracking individuals over time. The Principal Investigator will be able to view the identifiable information and data warehousing and research analytics will maintain a crosswalk between the actual identifiers and the proxy unique identifiers. Data quality checks will be conducted by Aurora with help from MIT to ensure that the randomization takes place as planned.

This data pull does not affect clinical decisions.

**STATISTICAL PLAN**

The main analysis will compare outcomes for providers who were randomized to receive CDS compared to those who were randomized to the control group using regression analysis. MIT will perform the data analysis. They will compute Intent-to-Treat estimates as our primary method.

Consider an outcome, Y , such as the number of non-advised scans ordered by each provider provider. For provider i, the estimating equation is:

$$Y_{i}=\beta_{0}+\beta_{1}{1(\text{Treatment})}_{i}+\beta_{2}X_{i}+\varepsilon_{i}$$

where Treatment_i is an indicator variable equal to one if the visiting provider was randomized to the treatment group and zero if the provider was randomized to the control group; X_i is a vector of control variables. These control variables should be uncorrelated with the treatment indicator, but they can aid in the precision of the estimate. For our main specification, we will include only the lagged dependent variable (defined during the quiet period prior to study launch) since this generates a first order improvement in power to estimate an effect of the CDS. As robustness checks, we will examine these regressions without any controls as well as controlling for the full set of observable provider characteristics.

$\beta_{1}$ is the parameter of interest and measures the Intent to Treat (ITT): the causal effect of a provider being randomized into the treatment group.

Sometimes the provider who orders the scan is not the same as the provider the patient initially visits. One robustness check will use the treatment status of the visiting provider as an instrument for the treatment status of the provider who orders the scan in order to compute Local Average Treatment Effect (LATE) estimates.

**Statistical Power**

Power simulations were conducted using two separate years of data for each provider (N=3506). We discovered that with this sample size and a 12-month study we would have the power to detect a 20% reduction in high-cost scans. This is similar to power calculations at another site, Mount Sinai Hospital in New York. Further, the rate of inappropriate scanning since the quiet period at Aurora began is on the order of 10%. At that rate, we will have the power to detect a 15% reduction in inappropriate scans, which is smaller than the effect sizes found in observational studies. In sum, we are powered to detect meaningful reductions in inappropriate scanning.

**POTENTIAL RISKS AND BENEFITS**

**Risks for Subjects:**

We believe the research imposes minimal risk to subjects as described above in the “Minimal Risks Justification” section. Below is a summary for provider and patients referencing the minimal risk, followed by the description of the risk of data disclosure.

**Providers**

Providers maintain their authority over imaging and treatment plans. The CDS intervention is non-obtrusive and can be easily overriden by the provider. Indeed, to order a scan once a best-practice alert is shown will take two clicks whether the provider chooses a different scan or not. Provider participation in the study is voluntary and does not impact their employment. We anticipate no physical, psychological, economic, or social risks to either providers or patients as a result of this study.

**Patients**

The study presents minimal risk to the patient given that this study will be using data that Aurora collects as a part of its normal operations, no procedures will be performed on patients as a part of this study, and investigators will have no direct contact with patients as a direct result of the study. Furthermore, analysis will be performed on a Limited Dataset of medical records that will be securely protected according to a Data Use Agreement developed by Aurora and MIT.

We do not anticipate any adverse impacts on patients whose providers use the decision support tool designed to improve ordering behavior. Patients in the control group will receive usual care. Further, we have clinical equipoise: The effects of clinical decision support are as yet unproven by a randomized controlled trial. As Aurora, and indeed CMS, propose implementing decision support for all providers, it is responsible and ethical to rigorously evaluate the impacts of this technology on scan orders, as compared to the status quo.

We are not specifically targeting any patient sub-population. We are excluding pediatric patients from this study.

**As a result, data disclosure is the primary risk and one that we take very seriously as described below.**

**Data Disclosure Risk**

All data shared outside of Aurora will conform to a De-identified Data Set as specified in the Privacy Rule.  Surrogate provider and patient identifiers will be provided.  Only data that Aurora normally collects through its normal operations will be collected through Aurora.

No identifiable health information will be newly created as a result of this research study. The risk of disclosure is incredibly small, as no identifiers will be released to external parties for research purposes.

**MIT Research Team’s Experience with Data Security**

MIT’s research team has extensive previous and ongoing experience with using and managing confidential data, including private health information such as data from Oregon Medicaid, Medicare claims data, and a very similar study at Mount Sinai in New York (among other health data).

The MIT Economics Department maintains a Unix/Linux-based research computing environment for its students and faculty members.  The research computing systems utilize enterprise-level hardware and are managed by a dedicated staff of IT professionals.  The Department leverages additional resources provided by MIT centrally, such as network infrastructure and professional co-location services in MIT's datacenters.  Department IT staff fully support private research servers purchased by individual faculty members.  This support includes account management, security patching, software installation and host monitoring. Secure servers will be utilized for the purposes of processing and analyzing data used in research projects.  All computations and analytical work will be performed exclusively on these servers.  File based permissions will be set to restrict data access to the research team.

All project data will be stored on a network attached storage (NAS) device.  A dedicated volume will be created on the NAS for exclusive storage of all data related to this research project.  Data on this volume will be served using the NFSv4 protocol and restricted to authorized hosts and users using IP-based host lists and Kerberos credentials. Kerberos credentials are assigned to specific individuals who are associated with MIT. Data on this volume will be accessible only to authenticated users on the project servers.  Data is backed-up to a secondary NAS device which is accessible only by IT personnel.

All network traffic is encrypted using the SSH2 protocol.  A Cisco Virtual Private Network (VPN) provides an additional level of encryption/access restriction for off-campus connections.  All server logins require two forms of authentication, a password and an SSH key pair. SSH Inactivity Timeout is used as the session timeout protocol.

**Waiver of Authorization Request**

Our understanding is that the following three criteria must be satisfied for an IRB or Privacy Board to approve a waiver of authorization under the Privacy Rule:

1. *The use or disclosure of protected health information involves no more than a minimal risk to the privacy of individuals, based on, at least, the presence of the following elements:*
   - *an adequate plan to protect the identifiers from improper use and disclosure;*

MIT and Aurora have data security procedures in place, and MIT has an excellent track record from previous studies using limited, de-identified, or identified data. Further, no directly identifying information will be shared with MIT researchers, thus protecting the identifiers from improper use and disclosure.

- - *an adequate plan to destroy the identifiers at the earliest opportunity consistent with conduct of the research, unless there is a health or research justification for retaining the identifiers or such retention is otherwise required by law; and*

No directly identifying information will be shared with MIT researchers, thus eliminating the need to destroy such identifiers.

- - *adequate written assurances that the protected health information will not be reused or disclosed to any other person or entity, except as required by law, for authorized oversight of the research project, or for other research for which the use or disclosure of protected health information would be permitted by this subpart;*

1. *The research could not practicably be conducted without the waiver or alteration; and*
2. *The research could not practicably be conducted without access to and use of the protected health information.*

The research could not be practicably carried out in the absence of the waiver for patients.

- 1. This study will be implemented at the provider level. This leads an informed consent process for patients to be impracticable for the following reasons:
     1. We believe a part of the effect of the decision support may be from providers learning over time which types of scans are more or less appropriate in different clinical scenarios. For this learning to occur as it would outside of a trial or research perspective, providers must have the opportunity to view the CDS alerts for each patient – not just a subset who consent.
     2. The consent process is likely to cause increased administrative burden, and impede or delay the normal course of care, as the patient reviews the consent form.
     3. The characteristics of patients who consent to the study are likely to be systematically different from those who do not consent. This would bias the results of the research if consenters are more (or less) likely to have a condition for which a provider would order a more (or less) appropriate scan.
     4. It is technologically infeasible to de-activate the decision support software for patients who do not consent.

In addition, all patients at Aurora see providers who could place an order, and consenting all Aurora patients would be a monumental task and one that would result in the study not being performed. Second, when a provider in the treatment group orders a scan on a patient who did not consent, it would be impractical to alert NDSC not to provide the best practice alert in real time.

Information on utilization and underlying health characteristics are essential for the measurement of the outcomes of this study: scan ordering patterns and downstream health utilization. Only de-identified information will be shared with MIT. This de-identified information will be coded, and Aurora will maintain the code (explained in Data Collection Section).

**Publicly Available De-identified Data**

A separate modification will be submitted in the future. The purpose of the modification will be to describe the purpose and methodology by which the results from this study be made publically available. Transparency in research, including openly sharing data, plays a key role in making science credible, and this reason is one of the main purposes for this plan.

**REFERENCES**

1. Orszag PR. The Overuse, Underuse, and Misuse of Healthcare. Congressional Budget Office. 2008.
2. Medicare Payment Advisory Commission. Report to the Congress, Medicare Payment Policy. Mar 2013.
3. America's Health Insurance Plans. Ensuring Quality through Appropriate Use of Diagnostic Imaging. Jul 2008.
4. Rosenthal DI, Weilburg JB, Schultz T, Miller JC, Nixon V, Dreyer KJ, Thrall JH. Radiology order entry with decision support: initial clinical experience. J Am Coll Radiol. 2006 Oct;3(10):799-806.
5. Lehnert BE, Bree RL. Analysis of appropriateness of outpatient CT and MRI referred from primary care clinics at an academic medical center: how critical is the need for improved decision support? J Am Coll Radiol. 2010 Mar;7(3):192-7. Erratum in: J Am Coll Radiol. 2010 Jun;7(6):466.
6. Blackmore CC, Mecklenburg RS, Kaplan GS. Effectiveness of clinical decision support in controlling inappropriate imaging. J Am Coll Radiol. 2011 Jan;8(1):19-25
7. ACR Select. <http://www.acrselect.org/index.html>.
8. American College of Radiology. Appropriateness Criteria.

**DATA VARIABLE LIST**

Data variable list to be sent to MIT is attached.

1. We hoped to have the ability to default to the highest-scored, appropriate, alternative scan. However, system limitations and the presence of a number of ties for “most appropriate alternative” forced us to eliminate this element of the intervention design. [↑](#footnote-ref-1)
